# LRRK2 negatively regulates glucose tolerance via regulation of membrane translocation of GLUT4 in adipocytes

**DOI:** 10.1002/2211-5463.13717

**Published:** 2023-10-26

**Authors:** Fumitaka Kawakami, Motoki Imai, Yuki Isaka, Mark R. Cookson, Hiroko Maruyama, Makoto Kubo, Matthew J. Farrer, Makoto Kanzaki, Rei Kawashima, Tatsunori Maekawa, Shun Tamaki, Yoshifumi Kurosaki, Fumiaki Kojima, Kenichi Ohba, Takafumi Ichikawa

**Affiliations:** ^1^ Department of Regulation Biochemistry, Graduate School of Medical Sciences Kitasato University Sagamihara Japan; ^2^ Department of Health Administration, School of Allied Health Sciences Kitasato University Sagamihara Japan; ^3^ Regenerative Medicine and Cell Design Research Facility, School of Allied Health Science Kitasato University Sagamihara Japan; ^4^ Department of Molecular Diagnostics, School of Allied Health Sciences Kitasato University Sagamihara Japan; ^5^ Cell Biology and Gene Expression Section, Laboratory of Neurogenetics, National Institute on Aging National Institutes of Health Bethesda MD USA; ^6^ Department of Cytopathology, Graduate School of Medical Sciences Kitasato University Sagamihara Japan; ^7^ Department of Microbiology, School of Allied Health Science Kitasato University Sagamihara Japan; ^8^ Department of Neurology and Fixel Institute University of Florida Gainesville FL USA; ^9^ Graduate School of Biomedical Engineering Tohoku University Sendai Japan; ^10^ Department of Medical Laboratory Sciences, School of Allied Health Sciences Kitasato University Sagamihara Japan; ^11^ Department of Pharmacology, School of Allied Health Sciences Kitasato University Sagamihara Japan

**Keywords:** adipocyte, glucose tolerance, GLUT4, LRRK2, Rab GTPase

## Abstract

Epidemiological studies have shown that abnormalities of glucose metabolism are involved in leucine‐rich repeat kinase 2 (LRRK2)‐associated Parkinson's disease (PD). However, the physiological significance of this association is unclear. In the present study, we investigated the effect of LRRK2 on high‐fat diet (HFD)‐induced glucose intolerance using Lrrk2‐knockout (KO) mice. We found for the first time that HFD‐fed KO mice display improved glucose tolerance compared with their wild‐type (WT) counterparts. In addition, high serum insulin and leptin, as well as low serum adiponectin resulting from HFD in WT mice were improved in KO mice. Using western blotting, we found that Lrrk2 is highly expressed in adipose tissues compared with other insulin‐related tissues that are thought to be important in glucose tolerance, including skeletal muscle, liver, and pancreas. Lrrk2 expression and phosphorylation of its kinase substrates Rab8a and Rab10 were significantly elevated after HFD treatment in WT mice. In cell culture experiments, treatment with a LRRK2 kinase inhibitor stimulated insulin‐dependent membrane translocation of glucose transporter 4 (Glut4) and glucose uptake in mouse 3T3‐L1 adipocytes. We conclude that increased LRRK2 kinase activity in adipose tissue exacerbates glucose tolerance by suppressing Rab8‐ and Rab10‐mediated GLUT4 membrane translocation.

## Graphical abstract

LRRK2 is a protein kinase involved in the pathogenesis of Parkinson's disease. In this study, we demonstrated that lrrk2 knockout mice fed a high‐fat diet have improved glucose tolerance compared with wild‐type, that LRRK2 is highly expressed in adipose tissue, and that inhibition of LRRK2 promoted insulin‐dependent GLUT4 membrane translocation and glucose uptake in cultured adipocytes.

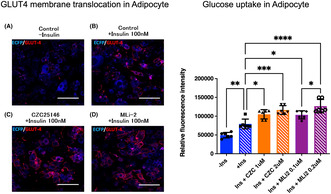

**Abbreviations**
AUC: area under the curve
GLUT4: glucose transporter 4
GSV: GLUT4 storage vesicles
HMW: high molecular weight
KRPH: Krebs‐Ringer phosphate‐Hepes
LRRK2: leucine‐rich repeat kinase 2
OGTT: oral glucose tolerance test
PD: Parkinson's disease
PM: plasma membrane
RIPA: radioimmunoprecipitation assay

Leucine‐rich repeat kinase 2 (LRRK2) is a protein kinase involved in the development of autosomal dominant Parkinson's disease (PD) and is the causative gene product of the PARK8 locus, first identified in 2002 [1, 2]. The LRRK2 protein contains, from N to C terminus, armadillo repeats (ARM), ankyrin repeats (ANK), leucine‐rich repeat (LRR), Ras‐of‐complex (ROC) GTPase, C‐terminal of ROC (COR), protein kinase, and WD40 domains. LRRK2 is expressed in neuronal and immune cells, and it is predicted to be a cytosolic protein because it does not contain a transmembrane domain [3, 4, 5, 6]. However, LRRK2 has been suggested to also localize to organelle membranes [7, 8], including endoplasmic reticulum, Golgi apparatus, early endosomes, lysosomes, synaptic vesicles, mitochondria, and plasma membrane [9, 10] depending on stimulus. Multiple physiological functions of LRRK2, including neurite outgrowth, apoptosis, autophagy, regulation of cytokine production, and membrane transport, have been reported [7, 10].

Recently, Rab GTPases have been reported as novel substrates for LRRK2 [11]. Two known LRRK2 substrates, Rab8a and Rab10, play an important role in insulin‐dependent membrane transport of glucose transporter 4 (GLUT4) [12]. GLUT4 is encapsulated in GLUT4 storage vesicles (GSV: GLUT4 storage vesicles) via the trans‐Golgi network and transported to the cell membrane by insulin signaling [12]. Binding of insulin to its receptor sequentially activates downstream targets, including IRS‐1, PI3‐K, and AKT. Activated AKT induces the membrane transport of GLUT4, which promotes glucose uptake in insulin‐sensitive tissues (adipose, muscle, and liver) [12]. It is also known that AMPK enhances GLUT4 expression and membrane transport. In adipocyte and muscle cells, when AMPK is activated by adiponectin or exercise stimulation, the Rab GTPase‐activating protein AS160 (gene name *TBC1D4*) is inhibited by AMPK‐mediated phosphorylation. As a result, Rab8a or Rab10 converts to a GTP‐binding active form, and these Rab GTPases induce the membrane transport of GLUT4 vesicles [13, 14]. Recently, it was reported that phosphorylation of AS160 is increased in LRRK2 deficient fibroblasts of aged KO mice [15]. It is therefore possible that LRRK2 may regulate membrane transport of GLUT4 via Rab8a and Rab10.

Recently, we reported a novel role of LRRK2 in glucose metabolism using dexamethasone (DEX)‐induced abnormal glucose tolerance model mice. Specifically, we found that KO mice improved abnormal glucose tolerance, even after treatment with DEX [16]. However, the role of LRRK2 on abnormalities in glucose tolerance caused by lifestyle‐related model such as HFD is unknown. Here, we find that Lrrk2 KO mice are relatively resistant to insulin‐related abnormalities on a HFD. Furthermore, we found that effects of LRRK2 inhibitor on glucose uptake and insulin‐dependent membrane transportation of GLUT4 in cultured adipocytes correlate with the phosphorylation of Rab8a and Rab10, suggesting a mechanistic link between LRRK2 activity and insulin responsiveness.

## Materials and methods

### Animal experiments

Five‐week‐old C57BL/6J male wild‐type (WT) and Lrrk2 exon 41‐KO mice (KO) [17] were used in this study. Only male mice were used in this study because epididymal adipose tissue was used to analyze protein expression and phosphorylation in adipocytes. Mice were allowed *ad libitum* access to either a normal diet (ND, AIN‐93M: 15% kJ from fat) or high‐fat diet (HFD, HFD‐60: 60% kJ from fat; obtained from Oriental Yeast Co., Ltd., Tokyo, Japan) with free access to water for 5 months. Nutritional composition of these diets is indicated in Table S1. There were therefore four experimental groups—WT‐ND (WT mouse fed ND), WT‐HFD (WT mouse fed HFD), KO‐ND (KO mouse fed ND), and KO‐HFD (KO mouse fed HFD). These mice were bred and maintained under an environment of room temperature 22 °C, 12‐h light and dark cycle in the SPF breeding room of the Kitasato University School of Medical Hygiene and Animal Experiment Facility, with three animals per cage.

### Oral glucose tolerance test (OGTT)

OGTT was performed at 1, 3, and 5 months after exposure to normal or HFD. After fasting by removing animals from each diet for 16 h from the day before the test, blood was collected from the tail vein of the mouse and fasting blood glucose measured using a glucometer (Glutest Neo Alpha; Sanwa Chemical Institute, Nagoya, Japan). Subsequently, a 16% glucose solution was orally administered with a gavage to a dose of 2 g·kg^−1^ body weight. Blood was collected from the tail vein at 15, 30, 45, 60, 90, and 120 min after the administration of glucose and the blood glucose level was measured as above. Glucose tolerance evaluation in OGTT was evaluated by the area under the curve (AUC) from the change in blood glucose concentration after oral administration of 16% glucose. Then, euthanizing and tissue extraction were performed in daylight 1 week after the OGTT [16].

### Measurements of plasma level of insulin at OGTT

For measurement of insulin levels at OGTT, mouse blood was collected separately from the blood glucose level measurement at 0 (fasting), 30, and 60 min at the OGTT into 150 μL of heparin solution as an anticoagulant to a final concentration of 50 μg·mL^−1^. The mixed heparin solution was allowed to stand on ice for 2 h, then centrifuged at 1000 ***g*** for 5 min at 4 °C. The collected plasma was cryopreserved at −80 °C until used for experiments. Plasma insulin levels were measured using LBIS mouse insulin ELISA kit (U‐type for ND group, T‐type for HFD group; FUJIFILM Wako Shibayagi Co., Shibukawa, Japan), following the manufacturer's instructions.

### Measurements of serum level of insulin, leptin, HMW‐adiponectin, TNF‐α, and IL‐6

We measured insulin, leptin, HMW‐adiponectin, and TNF‐α levels in mice serum after 5 months of feeding ND or HFD. Serum insulin, leptin, HMW‐adiponectin, and TNF‐α levels were measured using LBIS mouse insulin ELISA kit (T‐type), LBIS mouse leptin ELISA kit, Levis mouse/rat high‐molecular‐weight adiponectin ELISA kit, LBIS mouse TNF‐αELISA kit (FUJIFILM Wako Shibayagi Co.), and mouse IL‐6 ELISA kit (Proteintech, Rosemont, IL, USA), respectively, following the manufacturer's instructions.

### Western blotting analysis

Mouse tissue or cultured cell pellets were lysed in RIPA Buffer [(25 mm Tris–HCL (pH 7.5), 150 mm NaCl, 1% NP‐40, 1% Sodium Deoxycholate, 0.1% SDS with Halt TM Protease & Phosphatase Inhibitor Cocktail 100× (Thermo Scientific, Waltham, MA, USA) and 0.5 m EDTA solution (Thermo Scientific)] by sonication. Samples were centrifuged (14 000 ***g***, 4 °C, 15 min), the supernatant collected, mixed in a ratio of 3 : 1 with 4× sample buffer mixed with 25 : 1 of NuPAGE^®^ LDS sample Buffer 4× (Thermo Scientific) with 8 m dithiothreitol, and heated at 100 °C for 5 min. Protein concentrations were measured using the Pierce 660 nm Protein Assay Reagent (Thermo Scientific). Proteins were resolved by SDS‐polyacrylamide gel electrophoresis using 5–20% acrylamide gradient gels (ATTO, Tokyo, Japan). Separated proteins in the gel were transferred to a PVDF membrane (Merck Millipore, Burlington, MA, USA) using a Trans‐Blot Turbo Transfer System (BIO‐RAD, Hercules, CA, USA) and blocked with PVDF Blocking Reagent (TOYOBO, Osaka, Japan). After overnight incubation at 4 °C with primary antibody diluted with Can Get Signal^®^ solution 1 (TOYOBO), blots were washed three times with TBS‐T. Blots were then incubated with horseradish peroxidase‐conjugated or fluorescence‐conjugated secondary antibody diluted with Can Get Signal^®^ solution 2 (TOYOBO) for 1 h at room temperature. Membranes were washed again three times with TBS‐T and bands visualized using enhanced chemiluminescence (Pierce ECL Plus Substrate, Thermo Scientific) or detected by fluorescence imaging with an ODYSSEY imaging system (LI‐COR, Lincoln, NE, USA). To normalize the signal of phospho‐specific antibodies to the target protein, blots were stripped by incubating the blot with stripping buffer (62.5 mm Tris–HCl pH 6.8, 2% SDS, and 100 mm β‐mercaptoethanol) for 30 min at 70 °C, followed by wash steps with TBS‐T. Blots were then incubated with an antibody against the total target protein. Band signals were quantitatively analyzed with image studio software (LI‐COR) as described previously [16].

Antibodies against the following proteins were used: GLUT4 (2213S; Cell Signaling Technology, Danvers, MA, USA, 1 : 1000), AKT (9272S; Cell Signaling Technology, 1 : 2000), phospho‐AKT(Thr308, 4051S; Cell Signaling Technology, 1 : 1000), AMPK (2793S; Cell Signaling Technology, 1 : 2000), phospho‐AMPK (Thr172, 2531S; Cell Signaling Technology, 1 : 1000), E‐Cadherin (3195S; Cell Signaling Technology, 1 : 1000) β‐ACTIN (5125S; Cell Signaling Technology, 1 : 5000) and GAPDH (3683S; Cell Signaling Technology, 1 : 5000), GLUT1 (73015S; Cell Signaling Technology, 1 : 1000), LRRK2 (ab033474; Abcam, Cambridge, UK, 1 : 2000), phospho‐LRRK2 (Ser935, ab133450; Abcam, 1 : 1000), Rab8a (ab237702; Abcam, 1 : 4000), phospho‐Rab8a (Thr72, ab230260; Abcam, 1 : 1000), Rab10 (ab237703; Abcam, 1 : 4000), phospho‐Rab10 (Thr73, ab23026; Abcam, 1 : 1000), AS160 (ABV10742; ABGENT, San Diego, CA, USA, 1 : 2000), and phospho‐AS160 (Thr642, PA080083; CUSABIO, Houston, TX, USA, 1 : 1000). The secondary antibodies were HRP donkey anti‐mouse IgG (H + L) antibody (1 : 5000) and HRP donkey anti‐rabbit IgG (H + L) antibody (Biolegend, San Diego, CA, USA) all at a dilution of 1 : 5000.

### 3T3‐L1 cell culture and differentiation to adipocyte

3T3‐L1 cells obtained from RIKEN BioResource Center (Ibaraki, Japan) were cultured in low glucose (1 g·L^−1^) Dulbecco's modified Eagle's medium (DMEM) containing 10% fetal bovine serum and 5% (v/v) penicillin and streptomycin sulfate in a humidified atmosphere of 5% CO_2_ at 37 °C. For differentiation to adipocytes, cells were seeded plates or coverslip with high glucose (4500 mg·L^−1^) DMEM and 10% FBS with media change every 2–3 days until confluency reached 70%. Subsequently, cells were treated with 1.0 μm dexamethasone, 0.5 mm isobutyl methylxanthine (IBMX), and 1 μg·mL^−1^ bovine insulin in DMEM containing 10% FBS for 2 days without medium change and then 0.1% insulin‐containing fresh DMEM containing 10% FBS for the next 2 days. Thereafter, cells were maintained in insulin‐free DMEM with 10% FBS till the cells were completely differentiated into adipocytes (8 days after initiation) as described previously [16, 18].

### Immunofluorescent cytochemistry (ICC)

Myc‐GLUT4‐ECFP stably expressing 3T3‐L1 (3T3‐L1‐G4) cells were cultured and differentiated in 24‐well culture plates on Poly d‐lysine/Laminin‐coated coverslips as described previously [18, 19]. Cells were fixed in 4% paraformaldehyde in PBS and nonspecific immunoreactivity blocked using 1% BSA in PBS. Primary antibody solution (anti‐myc mouse monoclonal IgG in blocking solution; 1 : 300 dilution) was added to wells and incubated overnight at 4 °C. After washing, secondary antibody (Alexa 594 labeled anti‐mouse IgG) was used to detect the myc epitope of myc‐GLUT4‐ECFP located at the cell surface. After subsequent washing and mounting, a confocal fluorescence microscope was used to visualize stained cells. [16, 18].

### Glucose cellular uptake measurement in 3T3‐L1 cells

Glucose uptake in 3T3‐L1 cells was measured using a glucose cellular uptake measurement kit (COSMO) as described in the manufacturer's instructions. Briefly, differentiated 3T3‐L1 cells seeded on 12‐well plates were treated with LRRK2 inhibitors (1–2 μm CZC25146 or 0.1–0.2 μm MLi‐2) for 6 h. After culture medium was removed, cells were incubated in serum‐free medium for 6 h and washed with Krebs‐Ringer phosphate‐Hepes (KRPH) buffer alone and then KRPH buffer containing 2% BSA. After stimulating the cells with 100 nm insulin, 2‐deoxy glucose solution was added to 1 mm and incubated for 10 min. Then, cells were washed with cold PBS, and cells were collected with sample diluent buffer and immediately sonicated. Cell lysates were heated at 80 °C for 15 min and then immediately centrifuged (15 000 ***g***, 20 min). Supernatants were mixed with reaction solution containing fluorescent substrate and enzyme (diaphorase) in a 96‐well black plate and incubated at 37 °C for 2 h in the dark. The fluorescence intensity of the sample was measured by a fluorescence plate reader [16].

### Fractionation of plasma membrane from mouse adipose tissue or differentiated 3T3‐L1 cells

The plasma membrane (PM) was fractionated using a Minute PM Protein Isolation Kit (Invent Biotechnologies, Plymouth, MN, USA) according to the manufacturer's instructions. Briefly, adipose tissue or cell palette was homogenized with buffer A in the filter cartridge placed in 2 mL tube. After centrifugation at 16 000 ***g*** for 30 s and filtration through a filter cartridge, the filtered sample was centrifuged at 700 ***g*** for 1 min. The supernatant was collected and centrifuged at 16 000 ***g*** for 15 min. The pellet was resuspended in buffer B and centrifuged at 7800 ***g*** for 5 min. The supernatant was then centrifuged at 16 000 ***g*** for 30 min, and the pellet was collected as the PM fraction. The PM fraction was then used for GLUT4 quantification by western blot analysis [16].

### Data analysis

In animal experiments, data represent the mean ± standard error of the mean (SEM). In biochemical analysis, data represent the mean ± standard deviation (SD) of one of the three independent experiments each performed in triplicate. Data analysis and curve fitting were performed with graphpad prism 9 (GraphPad Software, Boston, MA, USA).

### Study approval

The animal experimental protocol was approved by the Animal Care and Use Committee (Approval number Eiken‐ken 18‐09‐04) and Genetic Modification Experiment Safety Committee (Approval number 3952) of the Kitasato University in agreement with accepted international standards and followed the recommendations in the ARRIVE guidelines. All experiments were performed in accordance with the relevant guidelines and regulations.

## Results

### LRRK2 deficiency is associated with lower weight gain after HFD

After 10 weeks of access to a HFD, KO mice gained significantly less weight than their WT counterparts, whereas no significant differences were observed between genotypes on ND (Fig. S1A,B). This did not appear to be due to differences in food consumption or body temperature between genotypes, as KO mice had higher intake than WT animals under either ND or HFD conditions (Fig. S1C,D), and there were no differences in body temperature between WT and KO mice (Fig. S2). Furthermore, it is reported that in open‐field exploration, both KO and WT mice showed no differences in walking distance or speed. While KO mice took longer rests, there was no significant difference observed across ages. The frequency of rests and short stops was consistent between both mouse types [17]. Thus, the suppression of body weight gain in HFD‐fed KO mice is presumably due to differences in metabolism between genotypes. In fact, some tissues, notably liver and adipose, weighed less in KO animals after HFD (Fig. S3A,B). Furthermore, serum triacylglycerol of HFD‐fed KO mice was significantly lower than that of WT animals (Fig. S4). These results suggest that endogenous LRRK2 may inhibit insulin‐sensitive tissue responsiveness to HFD‐induced weight gain and lipid uptake *in vivo*.

### LRRK2 knockout mice display improved glucose intolerance by HFD

**Fig. 1:**
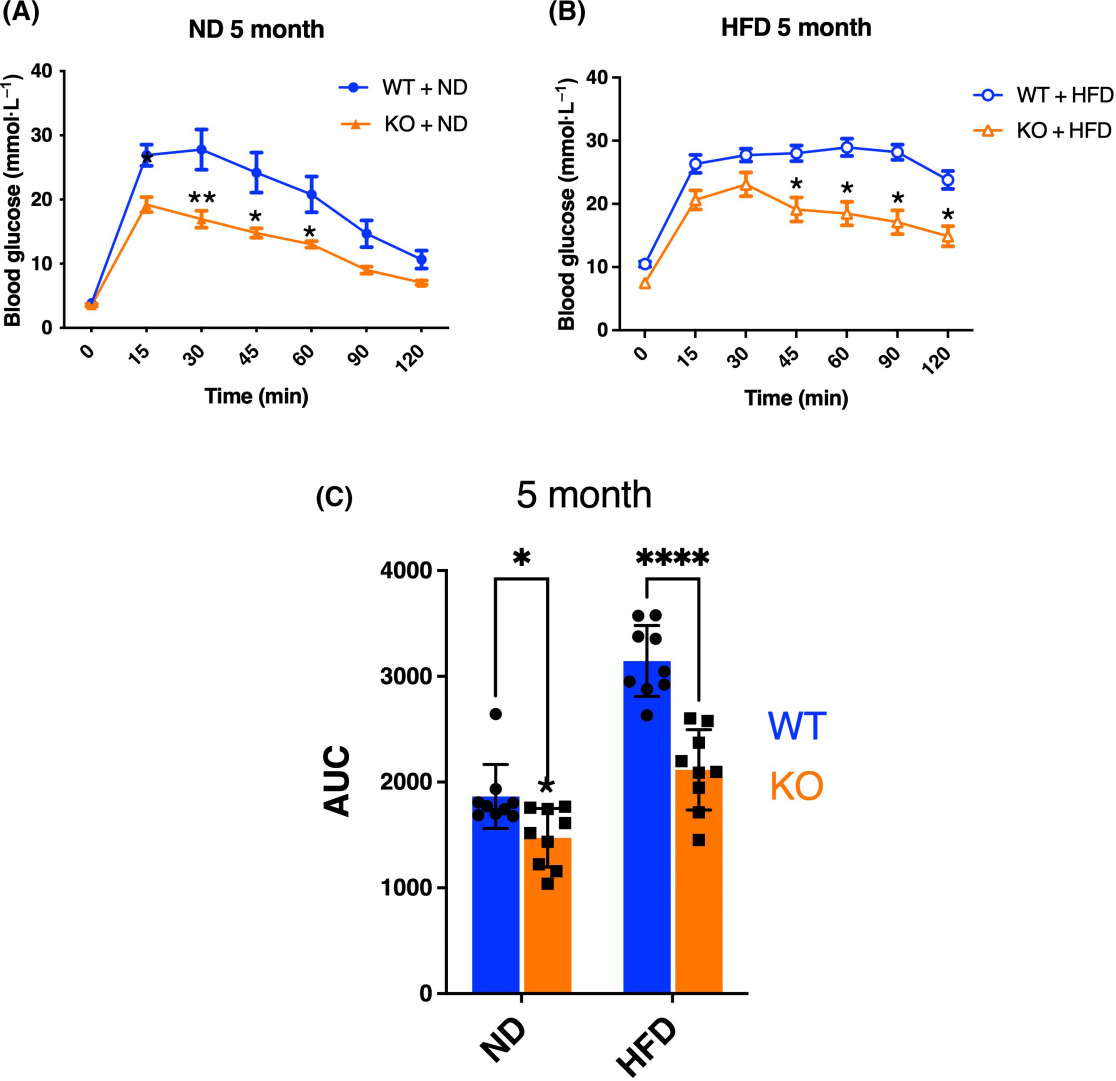
Comparison of blood glucose changes in OGTT of ND‐ or HFD‐fed WT and Lrrk2‐KO mice. Five‐week‐old WT and Lrrk2‐KO mice were reared on ND or HFD for 20 weeks. OGTT was performed at 5 months from the start of feeding each diet. Blood glucose variation curve of ND group (A) and HFD group (B) at 5 months are shown with wild‐type animals in blue and Lrrk2‐knockout in orange. Area under the curve (AUC) of OGTT at 5 months (C) was indicated. Data are presented as means ± SEM (*n* = 9 animals per group). Data of blood glucose variation curve were analyzed by two‐way ANOVA followed by Tukey's *post hoc* test. *P < 0.05; **P < 0.05; ****P < 0.0001 (WT vs. KO).

Based on these observations, we postulated that LRRK2‐knockout animals would show altered insulin responses, of which glucose tolerance is a widely used outcome measure. We therefore performed OGTT at 1, 3, and 5 months from the start of feeding ND or HFD in wild‐type and KO mice. In the ND group, the serum glucose level of KO mice after oral glucose was significantly lower than that of WT mice at each time point (Fig. 1A and Fig. S3A,B). After 1 month of HFD, the serum glucose levels of KO mice at the 15‐, 90‐, and 120‐min time points were significantly lower than that of WT (Fig. S5A). Furthermore, after 3 months of HFD, serum glucose was significantly lower in KO mice compared with WT at multiple tested time points (Fig. S5B). In the 5‐month HFD group, serum glucose level of KO mice was also significantly lower than that of WT mice (Fig. 1B). Additionally, the AUC for overall glucose responses of Lrrk2‐KO mice was significantly lower than that of WT at 1, 3, and 5 months in both normal and HFD (Fig. 1C and Fig. S5C,D).

### Alteration of serum insulin, leptin, high‐molecular‐weight adiponectin (HMW‐adiponectin), TNF‐α, and IL‐6

**Fig. 2:**
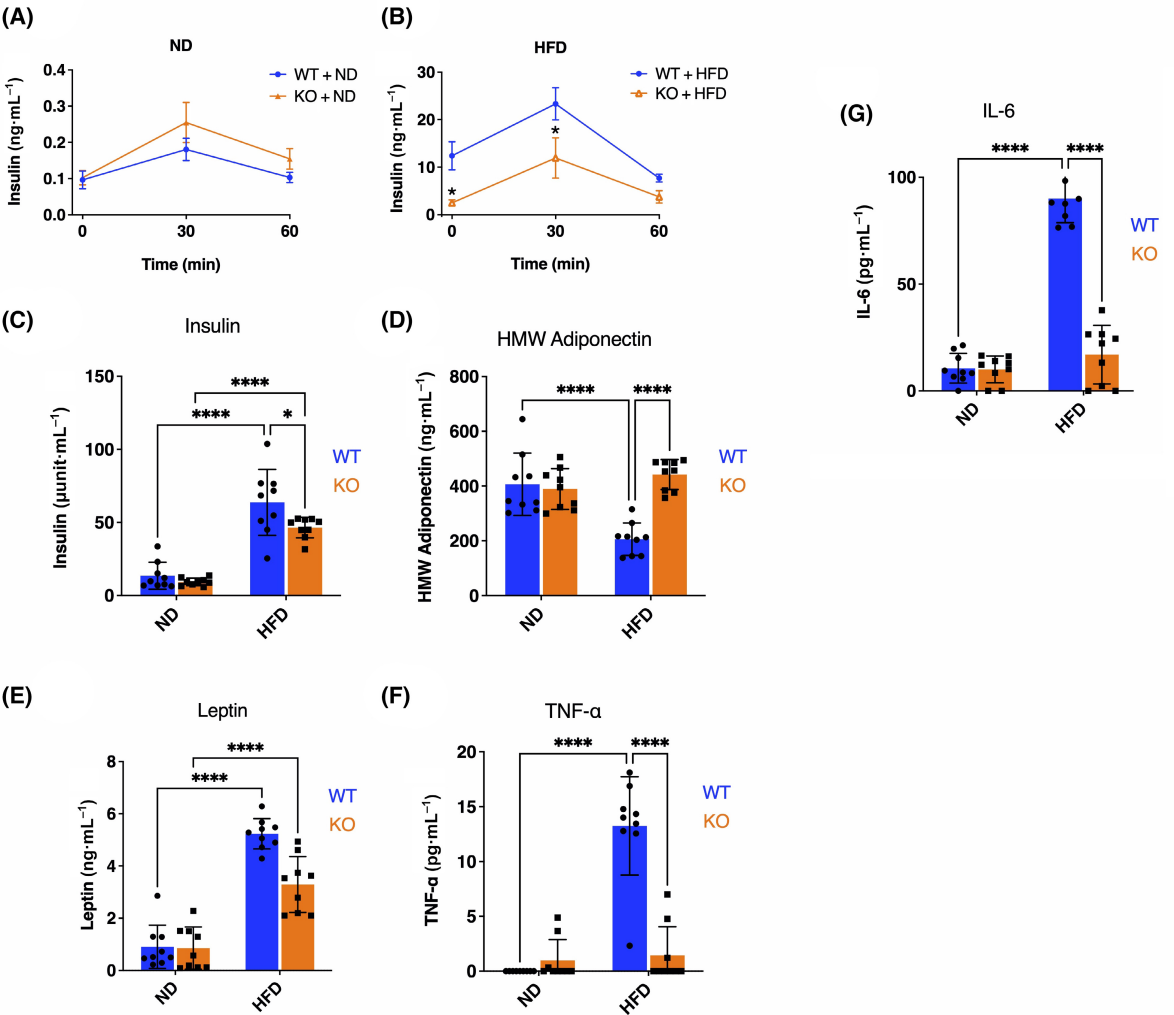
Comparison of serum insulin, leptin, HMW‐adiponectin, TNF‐α, and IL‐6 levels of ND‐ or HFD‐fed WT and Lrrk2‐KO mice. Time course of serum insulin levels in glucose injection at 5 months of ND‐fed WT and Lrrk2‐KO mice (A) and HFD‐fed WT and KO mice (B) was measured by ELISA. Data are presented as means ± SEM (*n* = 9 animals per group; each animal is shown by a single dot). Serum insulin (C), leptin (D) HMW‐adiponectin (E), TNF‐α (F), and IL‐6 (G) levels at 5 months of ND‐fed WT and Lrrk2‐KO mice and HFD‐fed WT and Lrrk2‐KO mice were measured by ELISA. Data are presented as means ± SEM (*n* = 9). The data were analyzed by two‐way ANOVA followed by Tukey's *post hoc* test. *P < 0.05; ****P < 0.0001 (WT vs. KO).

Next, we determined serum insulin, leptin, HMW‐adiponectin, TNF‐α, and IL‐6 after 5 months of exposure to ND or HFD. The fasting serum insulin level of HFD‐fed mice was 20‐ to 100‐fold higher than the ND group (Fig. 2A,B), confirming the expected hyperinsulinemia in our experimental condition. However, although no significant differences in insulin levels were noted under ND (Fig. 2A), the insulin level of HFD‐fed KO mice at baseline and at 30 min of OGTT was significantly lower than that of WT animals (Fig. 2B). We also found fasting glucagon levels were not different between WT and KO mice (Fig. S6). It is well known that glucose intolerance associated with high‐fat feeding leads to increased insulin and leptin, as well as decreased HMW‐adiponectin, in serum. We therefore assessed these markers in WT and KO mice. The results showed that insulin and leptin were significantly increased by HFD in both genotypes (Fig. 2C,D), but insulin was significantly lower in HFD‐fed KO than in WT mice (Fig. 2C). Leptin tended to be lower in KO mice on HFD than WT, but this difference was not statistically significant (Fig. 2D). HMW‐adiponectin level was significantly decreased by high‐fat diet feeding in wild‐type mice, but that there were no significant changes in LRRK2 KO mice (Fig. 2E).

Inflammatory conditions are known to be induced in HFD‐associated obesity. Here, we found that blood levels of the inflammatory cytokine TNF‐α and IL‐6 were markedly increased in HFD‐fed WT mice but not in the HFD KO mice (Fig. 2F,G). These biochemical assays support attenuation to the biological response in HFD‐induced obesity in LRRK2 knockout animals.

### Determination of phosphorylation and expression of LRRK2 and insulin signaling‐related molecules in the adipose tissue of ND‐ or HFD‐fed WT and KO mice

**Fig. 3:**
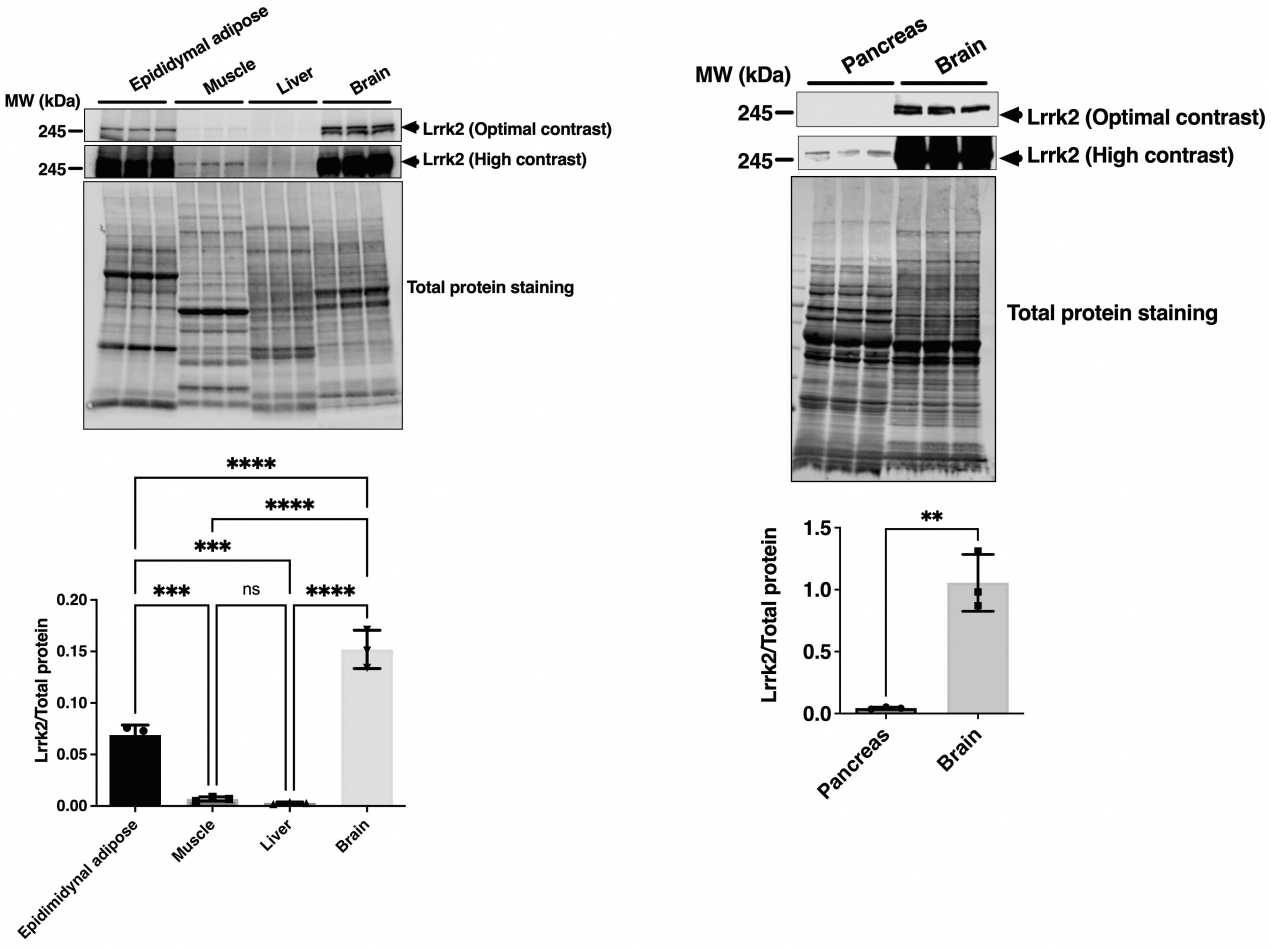
Detection of Lrrk2 protein expression in insulin‐relative tissues of WT mice. Lrrk2 protein expression in epididymal adipose, skeletal muscle, liver, pancreas, and brain of male WT mice was determined by western blot analysis, as described in Materials and methods. The band intensity of Lrrk2 was normalized by total protein amount. Data are presented as means ± SD (*n* = 3). The data were analyzed by one‐way ANOVA followed by Tukey's *post hoc* test. **P < 0.01; ***P < 0.001; ****P < 0.0001.

The above results indicate that KO mice have improved glucose tolerance and insulin resistance after exposure to the HFD, suggesting that LRRK2 may play an important role in glucose metabolism. However, this does not establish whether LRRK2 plays a direct role in insulin signaling within tissues or cells. Therefore, we investigated LRRK2 protein expression in insulin‐relative tissues, including adipose, skeletal muscle, liver, and pancreas in WT mice. Lrrk2 was highly expressed in adipose tissue, although slightly lower than in brain, while it was expressed at much lower levels in muscle, liver or pancreas (Fig. 3). These results suggest that if LRRK2 is involved in the whole animal response to HFD, then this is likely via effects on adipose tissue.

**Fig. 4:**
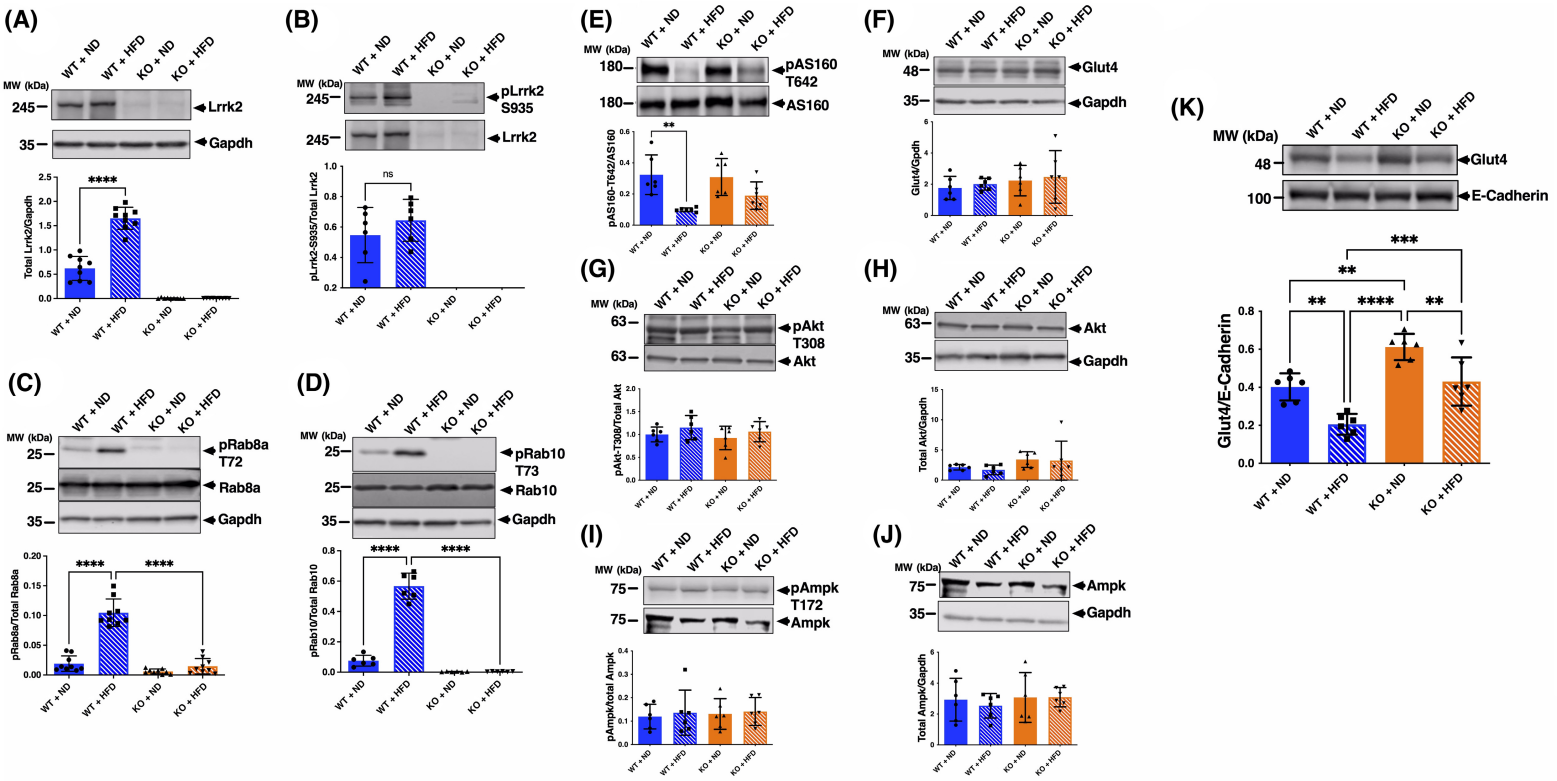
Comparison of protein expression and phosphorylation of Lrrk2, Rab8a and Rab10 in epididymal adipose tissue of ND‐ or HFD‐fed WT and Lrrk2‐KO mice. The expression of Lrrk2 (A), phospho‐Lrrk2 (Ser935) (B), phospho‐Rab8a (Thr72) (C), phospho‐Rab10 (Thr73) (D), phospho‐AS160 (Thr642) (E), GLUT4 (F), phospho‐Akt (Thr308) (G), total Akt (H), phospho‐Ampk (Thr172) (I), and total Ampk (J) was determined by western blot analysis. All proteins and GAPDH (as a loading control) were visualized by chemiluminescence and using an Odyssey Fc Dual‐Mode Imaging System (LI‐COR Biosciences, USA). The band intensity of Lrrk2, GLUT4, Akt, and Ampk were normalized by GAPDH. The intensity of phospho‐Lrrk2, phospho‐Rab8a and phospho‐Rab10, phospho‐AS160, phospho‐Akt, and phospho‐Ampk was normalized by their total protein band intensity. (K) Quantification of GLUT4 in the plasma membrane (PM) fraction obtained from adipose tissue of each mouse by western blotting as described in the Materials and methods. GLUT4 contents in PM fraction were determined by western blotting with an anti‐GLUT4 antibody. The intensity of Glut4 bands was normalized to E‐cadherin. Data are presented as mean ± SD (*n* = 6). The data were analyzed by one‐way ANOVA followed by Tukey's *post hoc* test. **P < 0.01; ***P < 0.001; ****P < 0.0001.

Next, we determined the effect of HFD on LRRK2 expression and kinase activity in adipose tissue. Rab8a and Rab10 are reported to be substrates for LRRK2 [21] and are also known to be important molecules for insulin signaling [12]. Therefore, we analyzed the phosphorylation of Rab8a and Rab10 as an indicator for LRRK2 kinase activity. Strikingly, we found that the expression of LRRK2 in adipose tissue was significantly increased in HFD‐fed WT mice (Fig. 4A). The same antibody gave no signal in KO mice, validating the utility of this reagent for evaluation of endogenous LRRK2. Additionally, whereas LRRK2 phosphorylation at Ser935 was not significantly changed, phosphorylation of Rab8a and Rab10 was both significantly increased in HFD‐fed WT mice (Fig. 4B–D). These results show that the endogenous expression and activity of LRRK2 are increased by chronic HFD *in vivo*.

Next, we investigated the expression of GLUT4 and expression or phosphorylation of AS160, AKT, and AMPK in adipose tissue of these mice by western blotting. We found that phosphorylation of AS160 was significantly decreased in HFD‐fed WT mice compared with ND‐fed WT mice (Fig. 4E). However, no significant difference was observed in expression and phosphorylation of Glut4, Akt, and Ampk (Fig. 4F–J). Furthermore, we found that the amount of Glut4 in the plasma membrane fraction (PM) from adipose tissue of KO mice was significantly higher than that of WT mice (Fig. 4K). In contrast, the expression level of Glut1 in the PM fraction was unchanged (Fig. S7). These results suggest that LRRK2 kinase activity may affect Glut4 membrane translocation but does not directly affect key insulin signaling‐related molecules.

### LRRK2 kinase inhibition promotes GLUT4 membrane translocation and glucose uptake in 3T3‐L1 adipocytes

Although the above results suggest that LRRK2 does not affect overall levels of GLUT4 in tissue, GLUT4 activity is also influenced by trafficking to the cellular membrane. To assess any potential effect of LRRK2 kinase activity on GLUT4 membrane translocation, we used 3T3‐L1 adipocytes stably expressing myc‐GLUT4‐ECFP (3T3‐L1‐G4). This GLUT4 vector has a c‐myc epitope tag in the first extracellular loop and enhanced cyan fluorescent protein (ECFP) at the C terminus, allowing us to detect the extracellular domain of GLUT4 translocated to the plasma membrane by immunostaining for myc tag without membrane permeabilization.

**Fig. 5:**
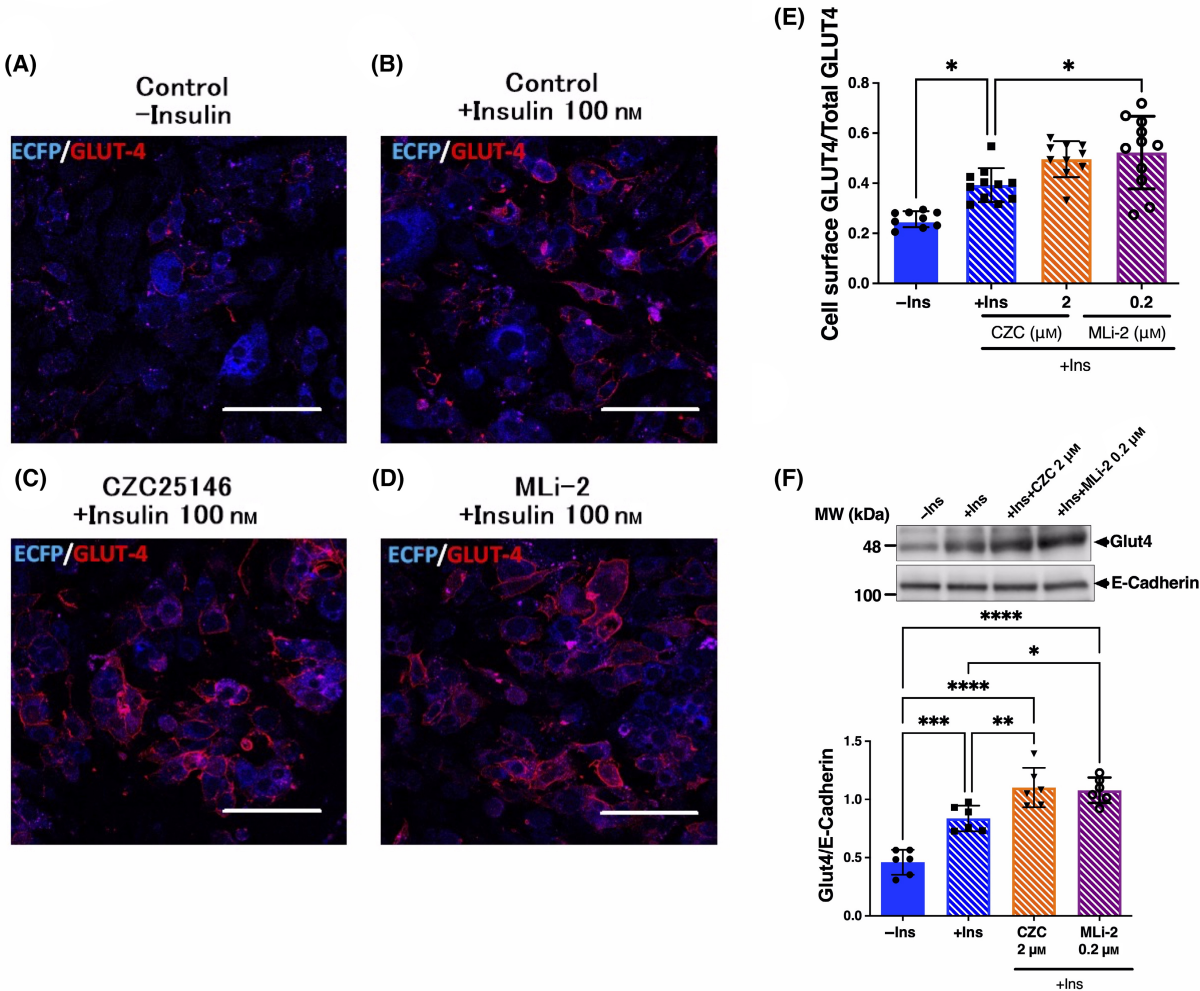
Effect of LRRK2 kinase inhibitor on the GLUT4 membrane translocation in adipocyte. Differentiated 3T3‐L1 adipocytes expressing Myc‐GLUT4‐ECFP were serum starved and then treated with or without CZC25146 (2 μm) and MLi‐2 (0.2 μm). Subsequently, cells were stimulated with insulin (100 nm) for 30 min. The cells were then fixed and stained with anti‐Myc antibody followed by Alexa594‐labeled secondary antibody. (A): control, (B): control + insulin, (C) CZC25146 (2 μm) + insulin, (D): MLi‐2 (0.2 μm) + insulin. (E) Quantification of GLUT4 on the cell surface was performed imagej by ImageJ (NIH, Bethesda, MA, USA) softwear. (F) Quantification of GLUT4 in the plasma membrane (PM) fraction obtained from differentiated 3T3‐L1 adipocyte after stimulation by 100 nm insulin with or without CZC25146 (2 μm) and MLi‐2 (0.2 μm) by western blotting as described in the Materials and methods. The intensity of GLUT4 bands was normalized to E‐cadherin. Data are presented as the mean ± SD (*n* = 6). The data were analyzed by one‐way ANOVA combined with Tukey's *post hoc* test. *P < 0.05; **P < 0.01; ***P < 0.001; ****P < 0.0001. Scale bar, 50 μm.

**Fig. 6:**
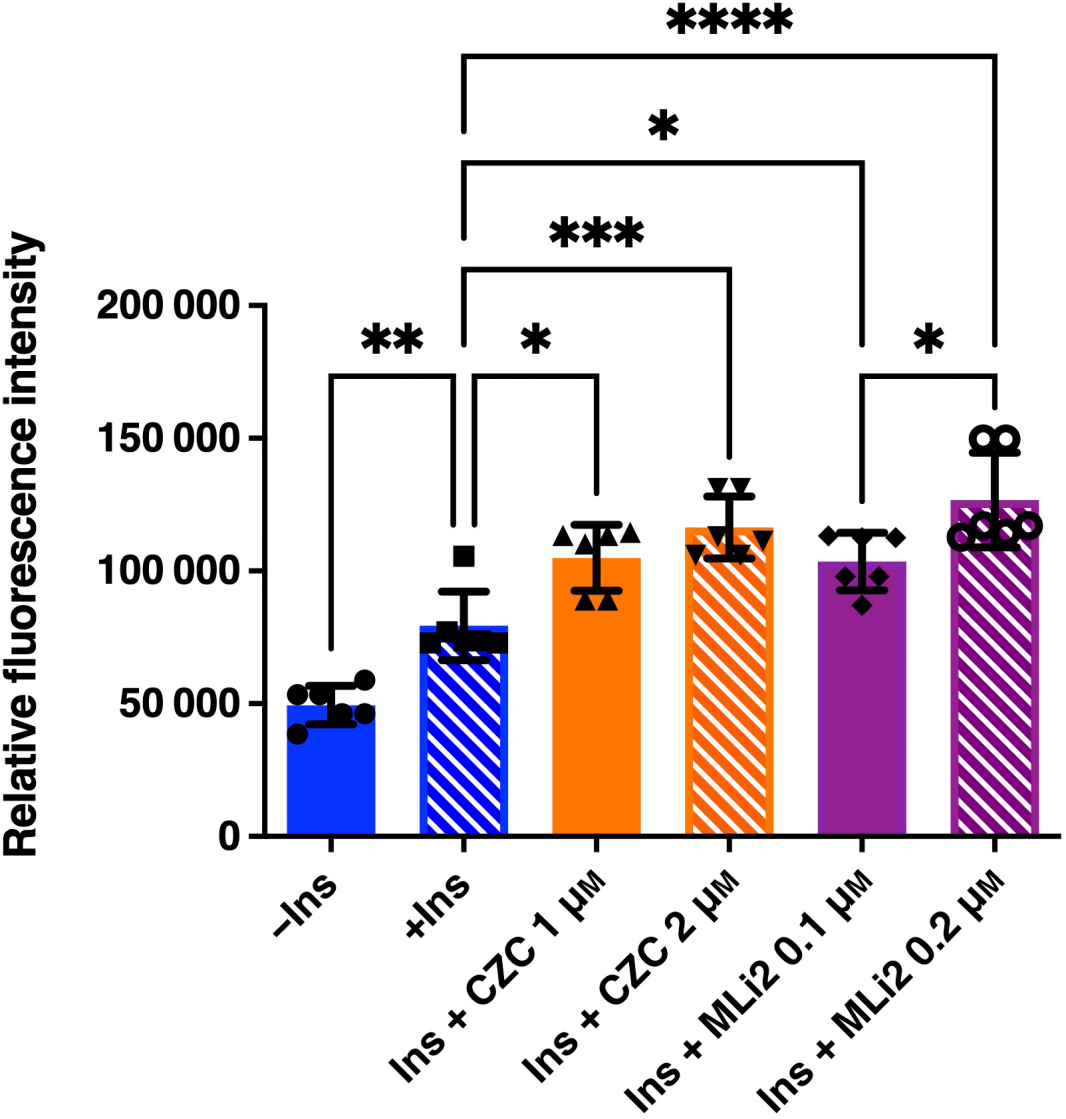
Effect of LRRK2 kinase inhibitor on the glucose uptake in adipocyte. Differentiated 3T3‐L1 adipocyte cells were preincubated with DMSO, CZC25146 (1 or 2 μm), or MLi‐2 (0.1 or 0.2 μm) incubation and were then treated with 100 nm insulin before treatment with 1 mm 2DG for 10 min. Subsequently, 2DG uptake into the cells was measured as described in Materials and methods. Data are presented as the mean ± SD (*n* = 6). The data were analyzed by one‐way ANOVA followed by Tukey's *post hoc* test. *P < 0.05; **P < 0.01; ***P < 0.001; ****P < 0.0001.

**Fig. 7:**
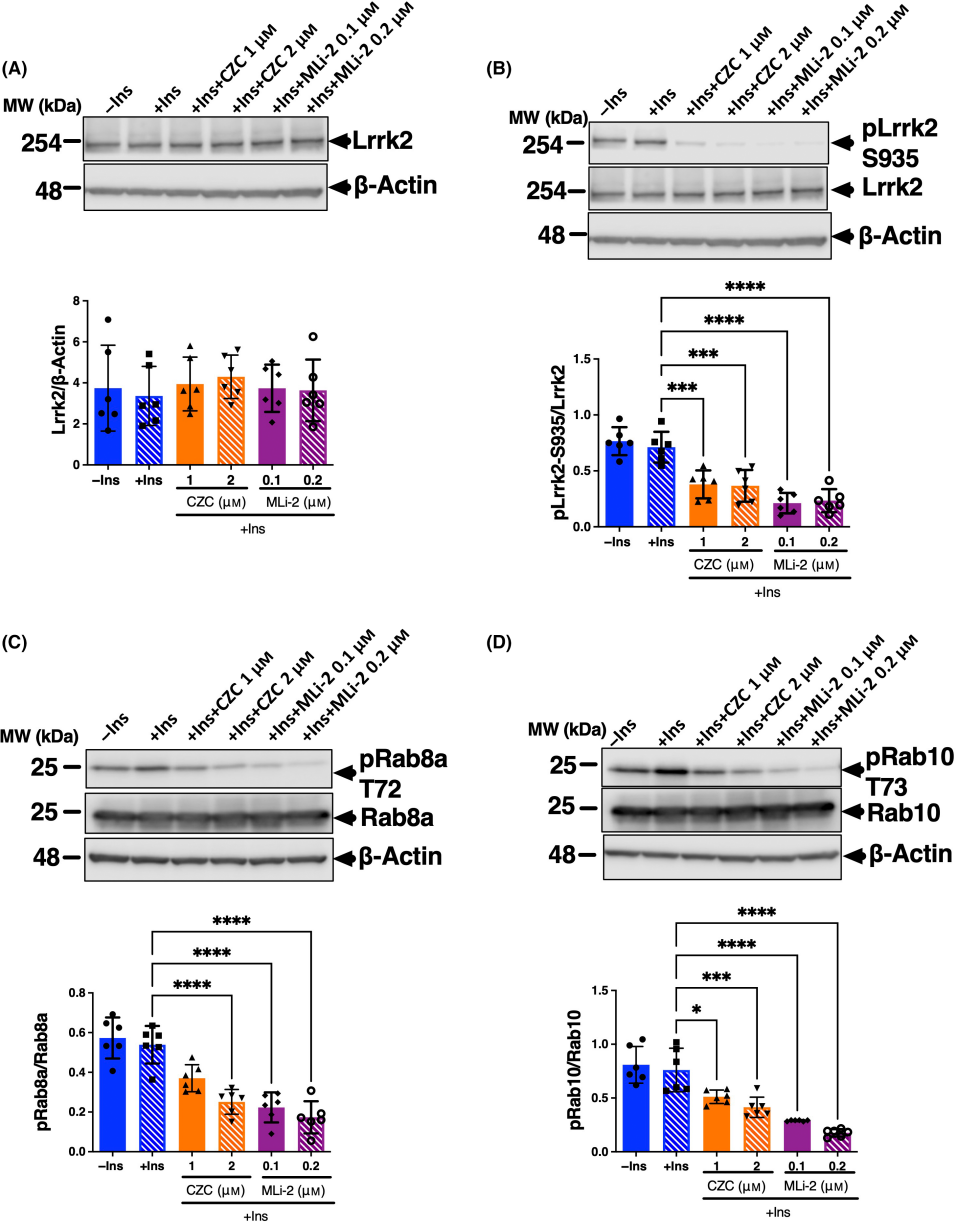
Effect of LRRK2 kinase inhibitor on the phosphorylation of Lrrk2, Rab8a and Rab10 in adipocyte. 3T3‐L1 cells treated with or without CZC25146 (1 and 2 μm) and MLi‐2 (0.1 and 0.2 μm) after serum starved and then stimulated with insulin for 30 min. The cells were harvested and analyzed by western blotting using antibodies against phosphorylated or total protein such as (A) Lrrk2, (B) phospho‐Lrrk2 (Ser935), (C) phospho‐Rab8a (Thr72), and (D) phospho‐Rab10 (Thr73). The expression level of phosphorylated protein was normalized against the total expression level of the target protein. Data are presented as means ± SD (*n* = 6). The data were analyzed by one‐way ANOVA combined with Tukey's *post hoc* test. *P < 0.05; ***P < 0.001; ****P < 0.0001.

Differentiated 3T3‐L1‐G4 cells were stimulated by insulin in the presence or absence of two structurally distinct LRRK2 kinase inhibitors, CZC25146 and MLi‐2. As expected, while there was no signal in the absence of insulin, the fluorescence intensity of myc staining was increased by insulin stimulation (Fig. 5A,B,E). Fluorescence intensity at plasma membrane was potently increased by the addition of both LRRK2 kinase inhibitors in the presence of insulin (Fig. 5C–E). Quantification of Glut4 protein amount in plasma membrane fraction by western blotting also confirmed that insulin‐dependent Glut4 membrane translocation is promoted by LRRK2 inhibitor (Fig. 5F). In contrast, no changes in the expression level of Glut1 in the PM fraction were observed (Fig. S8). Further supporting these observations, we also found that the same LRRK2 kinase inhibitors significantly promoted insulin‐dependent glucose uptake in normal 3T3‐L1 cells (Fig. 6). Phosphorylation of Lrrk2, Rab8a, and Rab10 was also significantly decreased under the same conditions of LRRK2 inhibition (Fig. 7B–D), whereas no effect on the Lrrk2 expression was observed (Fig. 7A).

In addition, no effect of LRRK2 inhibitors on insulin‐dependent phosphorylation of Akt was observed (Fig. S9A). Therefore, we speculate that LRRK2 is not directly involved in Akt signaling. In contrast, phosphorylation of T172 of Ampk was increased only with MLi‐2 treatment (Fig. S9B) but not with treatment with CZC25146, suggesting that inhibition of kinases other than LRRK2 by MLi‐2 may have increased the phosphorylation of Ampk at Thr172. Since Ampk phosphorylation was unchanged in adipose tissue from KO mice, it is unlikely that inhibition of LRRK2 increased Ampk phosphorylation. Furthermore, since Ampk activation is known to promote membrane trafficking of GLUT4, the synergistic effect of LRRK2 inhibition and Ampk activation in MLi‐2‐treated cells is expected to promote GLUT4 membrane trafficking. These results suggest that LRRK2 kinase activity negatively regulates membrane translocation of GLUT4 in adipocytes, probably through direct phosphorylation of Rab8a and Rab10, rather than through insulin signaling pathways involving Akt.

## Discussion

In the present study, we investigated the effect of LRRK2 on the regulation of blood glucose level using an abnormal glucose tolerance model evoked using a HFD. We found that the deterioration of glucose tolerance with HFD was significantly suppressed in KO mice. In addition, KO mice had lower serum insulin and leptin levels than WT mice treated with HFD, and the HFD‐induced decrease in serum adiponectin levels was also improved in KO mice. Furthermore, the increase in both blood TNF‐α and IL‐6 due to obesity was suppressed in KO mice. Our findings identify a new physiological role of LRRK2 that regulates glucose tolerance *in vivo*. However, since the present study did not evaluate overall activity and amount of exercise of WT and KO mice, additional experiments would be required to determine whether the observed suppression of body weight gain and glucose intolerance in KO mice under high‐fat diet (HFD) conditions can be attributed to exercise activity.

To understand the mechanism of LRRK2‐mediated regulation of blood glucose levels, we determined the expression of LRRK2 protein in insulin‐related tissues. We found that Lrrk2 was highly expressed in adipose tissues compared with other insulin‐related tissues such as pancreas, skeletal muscle, and liver. Adipose tissue is highly insulin‐responsive and plays a central role in regulation of the whole‐body energy and glucose homeostasis. In adipose tissue, insulin signaling induces glucose uptake by stimulation of membrane translocation of the insulin‐dependent glucose transporter GLUT4 [12]. Activation of AKT and AMPK signaling enhances the expression and membrane translation of GLUT4 by phosphorylation‐mediated inhibition of the Rab GAP AS160, which is an inhibitor for Rab8a and Rab10 on GLUT4 vesicles [12]. As these Rab GTPases are kinase substrates for LRRK2 [11], we determined the expression and phosphorylation of LRRK2, Rab8a, Rab10, and insulin signaling molecules such as GLUT4, AS160, AKT, and AMPK after HFD. Strikingly, we found that Lrrk2 expression and phosphorylation of Rab8a and Rab10 was significantly increased in adipose tissue of HFD‐fed WT mice.

Previous reports have shown that LRRK2 phosphorylates conserved residues located on the switch II domain of Rab substrates, and this phosphorylation causes each Rab to convert to an inactive, GDP‐bound, form [19]. In addition, the GTP/GDP‐bound state of Rabs is regulated by inherent GTPase activity, and Rab‐specific GTPase‐activating protein (GAP) is required for full activation [20]. AS160, known as GAP of Rab 2A, 8A, 10, and 14, regulates GLUT4 membrane translocation in adipocytes [20]. Previously, it has been shown that unphosphorylated AS160 binds to GLUT4 vesicles and inhibits GLUT4 membrane translocation and that AS160 phosphorylated by AKT or AMPK overcomes this inhibitory effect [20]. Thus, phosphorylation of AS160 is required for insulin‐stimulated translocation of GLUT4 to the plasma membrane in adipocytes. Here, we found that phosphorylation of AS160 in adipose tissue was significantly decreased by HFD in WT mice but not KO mice. In insulin‐resistant adipose tissue induced by HFD, Akt activity is suppressed and, as a result, there is a decrease in AS160 phosphorylation and upregulation of Rab GAP activity of AS160. Therefore, our results suggest that increased the phosphorylation of Rab8a and Rab10 due to enhanced LRRK2 expression and decrease in AS160 phosphorylation caused by HFD may synergistically downregulate GLUT4 membrane translocation in adipose tissue.

To confirm the suppressive effect of LRRK2 on the GLUT4 membrane translocation at a cellular level, we performed immunofluorescence analysis of GLUT4 using mouse 3T3‐L1 adipocyte stably expressing Myc‐GLUT4‐ECFP. It was found that both two structurally distinct LRRK2 kinase inhibitors (CZC25146 and MLi‐2) robustly promoted the membrane translocation of GLUT4. Furthermore, these two LRRK2 inhibitors significantly increased glucose uptake in the cells. These results suggest that LRRK2 kinase activity suppresses the insulin‐dependent glucose uptake through negative regulation of GLUT4 membrane translocation, likely via induction of the phosphorylation of Rab8a and Rab10 in adipocytes.

Using western blotting, we demonstrated that both LRRK2 inhibitors significantly inhibited the phosphorylation of LRRK2 (Ser935), Rab8a (Thr72), and Rab10 (Thr73) under insulin‐stimulated conditions, whereas insulin stimulation did not alter LRRK2 and Rab phosphorylation. In addition to this, we found that LRRK2 inhibition had no effect on the insulin‐dependent Akt phosphorylation. We therefore infer that insulin stimulation has no direct effect on LRRK2 kinase activity and Rab phosphorylation, and LRRK2 kinase activity does not directly affect insulin signaling in adipose tissue. In other words, insulin stimulation enhances GLUT4 translocation by increasing GTP‐bound Rab through inhibition of AS160, while LRRK2 inhibits GLUT4 translocation by increasing GDP‐bound Rab through structural transformation by Rab phosphorylation. These two actions may regulate the GTP/GDP binding state of Rabs and play a regulatory role in physiological GLUT4 membrane translocation. In summary, our results suggest that inhibition of LRRK2 and insulin stimulation synergistically promote GLUT4 membrane translocation by increasing GTP‐bound Rab.

Previously, it has been reported that both insulin‐stimulated glucose uptake and GLUT4 translocation to the plasma membrane are reduced by about half in adipocytes of Rab10 knockout mice, indicating that both Rab10‐dependent and Rab10‐independent pathways are involved in insulin‐dependent glucose uptake in adipose tissue [21]. Therefore, analysis of the association of LRRK2 with other Rabs is necessary to fully elucidate the role of LRRK2 in GLUT4 membrane translocation.

In addition, it is widely known that glucocorticoids, estrogens, catecholamines, diet, ketosis, excess energy, circadian rhythms, and inflammation are associated with regulation of blood glucose level [22, 23, 24, 25, 26]. As LRRK2 is highly expressed in the brain and in various immune cells [4], it is likely that LRRK2 is associated with different mechanisms than the Rab‐GLUT4 pathway outlined here. Additionally, in the present study, we observed elevated expression of LRRK2 in the adipose tissue of mice on a high‐fat diet. Our proposed interpretation is that this increased LRRK2 expression in adipocytes impedes the membrane trafficking of GLUT4. However, given that obesity induces infiltration of immune cells such as macrophages into adipose tissue, it will be crucial to discern whether the rise in LRRK2 expression in obese adipose tissue is intrinsic to adipocytes or a consequence of immune cell infiltration. A meticulous analysis involving immunostaining and flow cytometry is required to distinguish between these possible explanations. Moreover, in order to clarify whether LRRK2 is specifically involved in the regulation of glucose uptake in adipocytes, it will be necessary to analyze adipocyte‐specific LRRK2 knockout animals. Nevertheless, it seems certain that LRRK2 is involved in abnormal glucose tolerance, and hence, we propose that inhibition of the LRRK2 kinase activity targeted to the improvement of glucose intolerance may be a new strategy for the treatment of glucose metabolism disorders.

Finally, it has been reported that abnormalities of glucose metabolism are found in 50–80% of PD patients and that about 40% of patients with type 2 diabetes has high risk for developing PD (27–31). In addition, insulin resistance and pre‐diabetic condition are thought to adversely affect PD pathology, especially insulin resistance is known to worsen the pathology of PD and increase the risk of dementia [27, 28, 29, 30, 31]. Furthermore, statin therapy, a cholesterol‐lowering drug, delays the onset of Parkinson's disease in diabetic patients [32]; thus, it is suggested that type 2 diabetes and PD may share a common mechanism of pathogenesis. Our findings suggest that abnormal glucose metabolism caused by LRRK2 activation may be important in the pathogenesis of PD.

## Conflict of interest

The authors declare no conflict of interest.

### Peer review

The peer review history for this article is available at https://www.webofscience.com/api/gateway/wos/peer‐review/10.1002/2211‐5463.13717.

## Author contributions

FKa and TI conceived and designed the project; FKa, MI, YI, HM, MKu, MJF, MKa, RK, TM, ST, YK, FKo, and KO acquired the data; FKa and MI analyzed and interpreted the data; and FKa and MRC wrote the paper.

## Supporting information


**Fig. S1.** Effect of normal diet (ND) or high‐fat diet (HFD) on the body weight changes, food consumption, and tissue weight of WT and Lrrk2‐KO mice.
**Fig. S2.** Measurement of body temperature WT and Lrrk2‐KO mice.
**Fig. S3.** Effect of normal diet (ND) or high‐fat diet (HFD) on the tissue weight of WT and Lrrk2‐KO mice.
**Fig. S4.** Measurement of serum triacylglycerol level in ND‐ or HFD‐fed WT and Lrrk2‐KO mice.
**Fig. S5.** Comparison of blood glucose changes in OGTT of ND‐ or HFD‐fed WT and Lrrk2‐KO mice.
**Fig. S6.** Comparison of serum glucagon levels of ND‐ or HFD‐fed WT and Lrrk2‐KO mice.
**Fig. S7.** GLUT1 contents in PM fraction prepared from adipose tissue of ND‐ or HFD‐fed WT and Lrrk2‐KO mice.
**Fig. S8.** GLUT1 contents in PM fraction prepared from adipocyte.
**Fig. S9.** Effect of LRRK2 kinase inhibitor on the phosphorylation of Akt and Ampk in adipocyte.
**Table S1.** Nutritional composition of the normal and high‐fat diets fed to mice.

## Data Availability

The raw data presented in this study are available on request from the corresponding author.
