# Supplementary material for: LRRK2 negatively regulates glucose tolerance via regulation of membrane translocation of GLUT4 in adipocytes
Source: FEBS Open Bio. 2023 Oct 26;13(12):2200–14. doi: 10.1002/2211-5463.13717 (PMC10699104; doi:10.1002/2211-5463.13717)
Supplement: Supplementary file 1 — Fig. S1. Effect of normal diet (ND) or high‐fat diet (HFD) on the body weight changes, food consumption, and tissue weight of WT and Lrrk2‐KO mice. Fig. S2. Measurement of body temperature WT and Lrrk2‐KO mice. Fig. S3. Effect of normal diet (ND) or high‐fat diet (HFD) on the tissue weight of WT and Lrrk2‐KO mice. Fig. S4. Measurement of serum triacylglycerol level in ND‐ or HFD‐fed WT and Lrrk2‐KO mice. Fig. S5. Comparison of blood glucose changes in OGTT of ND‐ or HFD‐fed WT and Lrrk2‐KO mice. Fig. S6. Comparison of serum glucagon levels of ND‐ or HFD‐fed WT and Lrrk2‐KO mice. Fig. S7. GLUT1 contents in PM fraction prepared from adipose tissue of ND‐ or HFD‐fed WT and Lrrk2‐KO mice. Fig. S8. GLUT1 contents in PM fraction prepared from adipocyte. Fig. S9. Effect of LRRK2 kinase inhibitor on the phosphorylation of Akt and Ampk in adipocyte. Table S1. Nutritional composition of the normal and high‐fat diets fed to mice. [file FEB4-13-2200-s001.docx]

Supplemental information

 
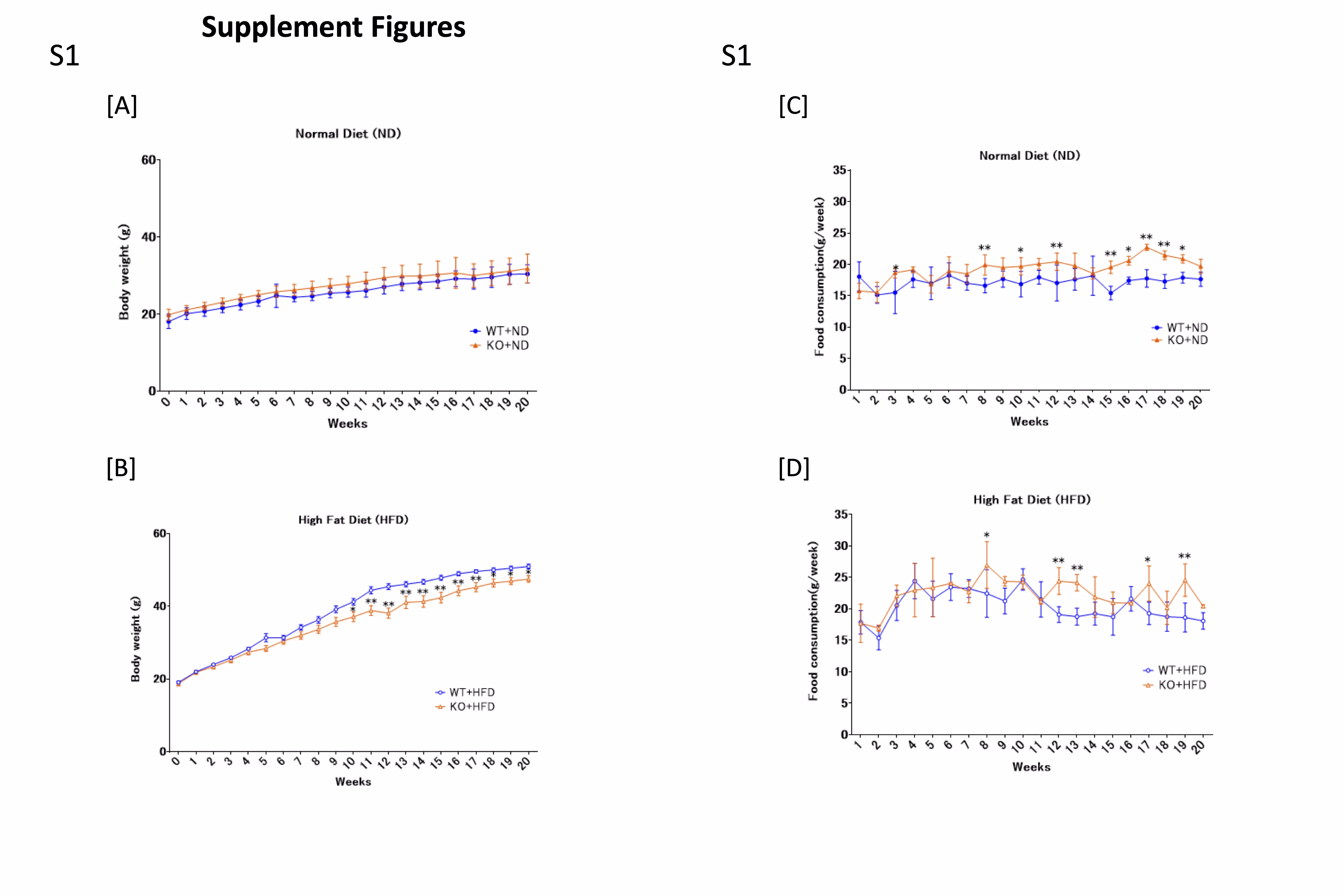


S1. Effect of normal diet (ND) or high-fat diet (HFD) on the body weight changes, food consumption and tissue weight of WT and Lrrk2-KO mice.

Five-week old WT and Lrrk2-KO mice were treated with ND or HFD for 20 weeks. We measured the body weight (A,B) and food consumption (C,D). Data are presented as means ± SEM (n = 9). The data were analyzed by two-way ANOVA combined with Sidak post hoc test. *p<0.05, **p<0.01 (WT vs Lrrk2-KO)


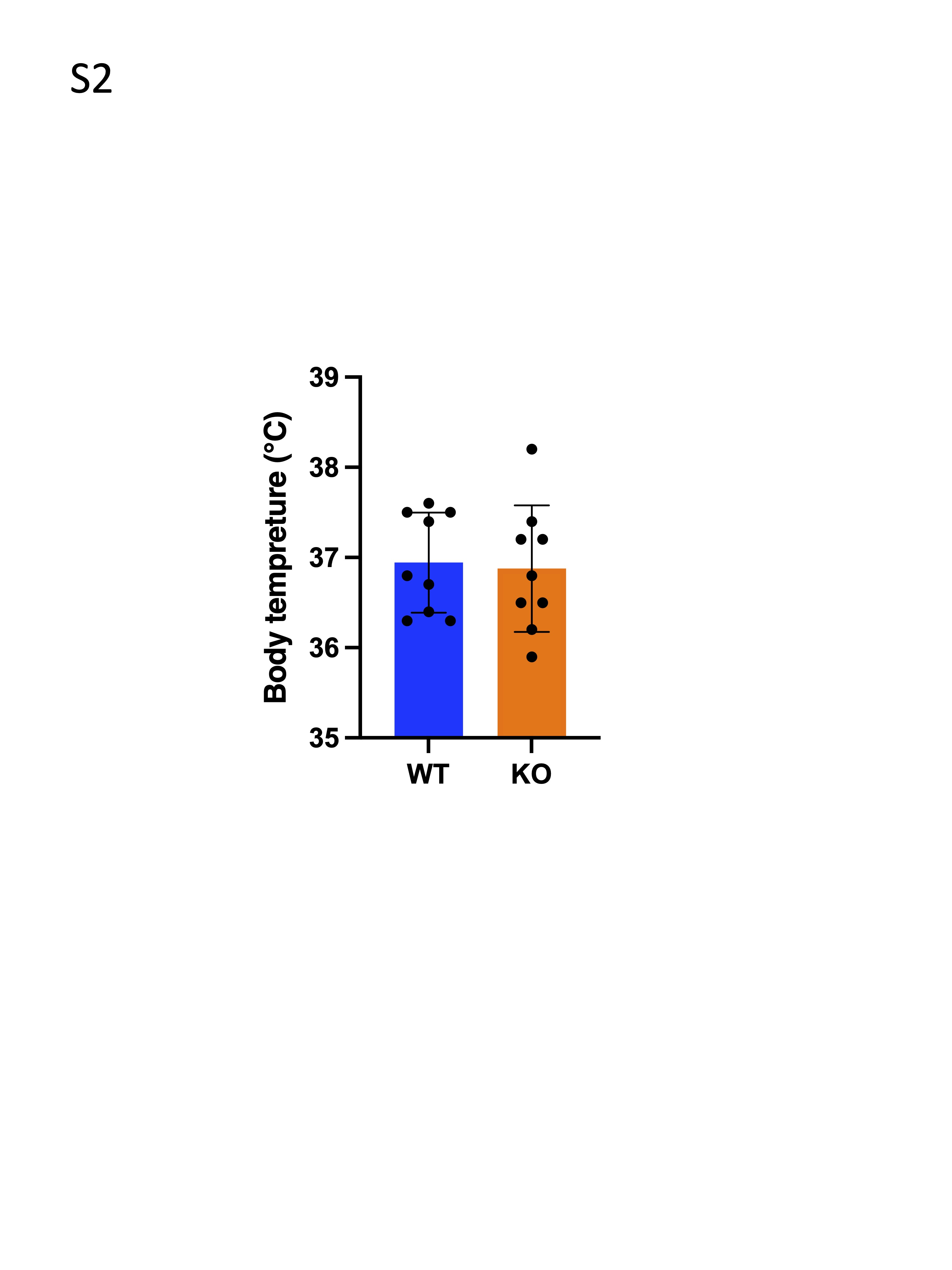


S2. Measurement body temperature WT and Lrrk2-KO mice

The probe of the thermometer was inserted into the mouse anus of the mice to measure their deep body temperature. Data are presented as means ± SEM (n = 9).


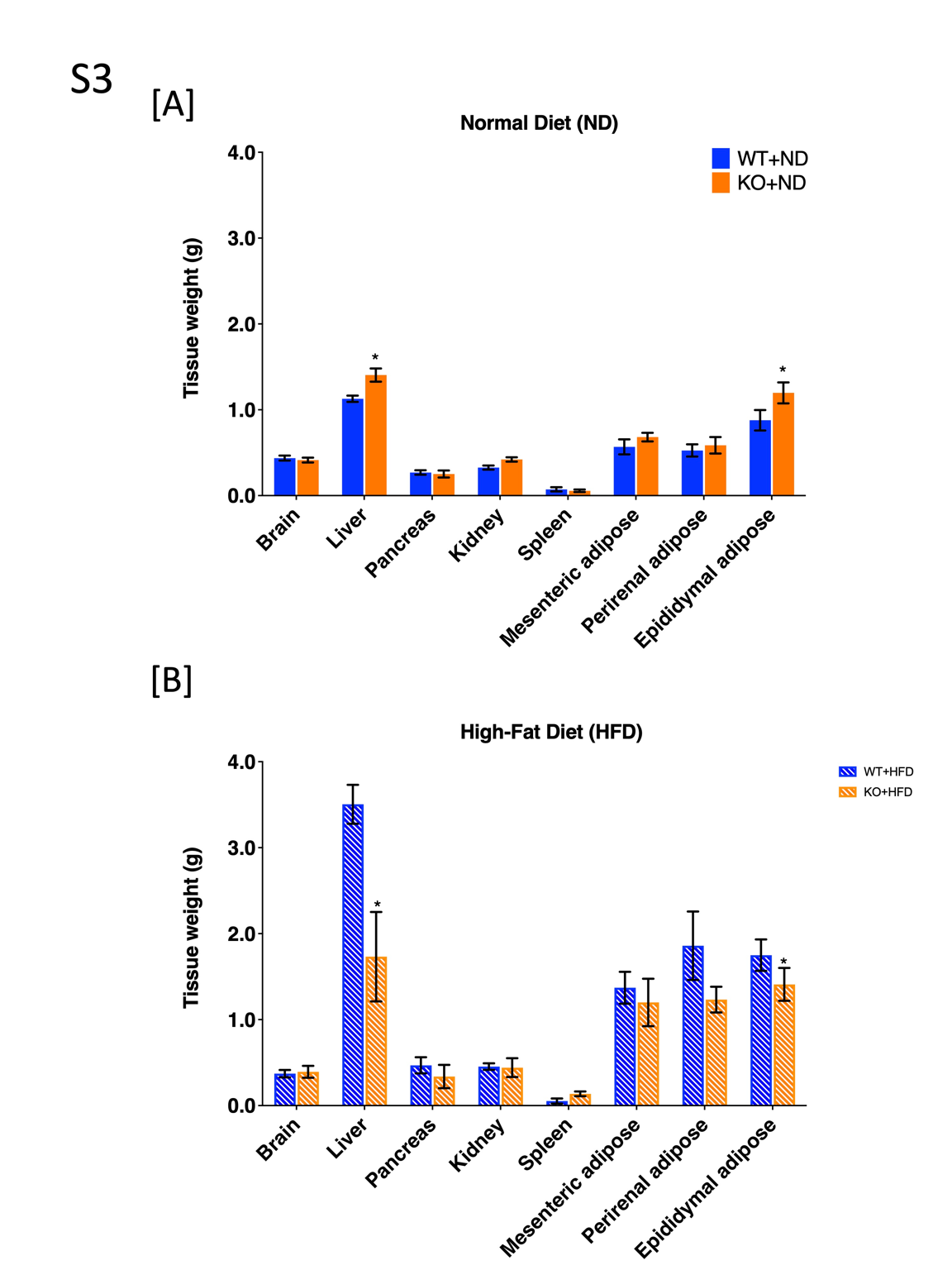


S3. Effect of normal diet (ND) or high-fat diet (HFD) on the tissue weight of WT and Lrrk2-KO mice.

Five-week old WT and Lrrk2-KO mice were treated with ND or HFD for 20 weeks. Mice were sacrificed at the end of week 20 and tissues were collected and weighed (A,B). Data are presented as means ± SEM (n = 9). The data were analyzed by two-way ANOVA combined with Sidak post hoc test. *p<0.05, **p<0.01 (WT vs Lrrk2-KO)


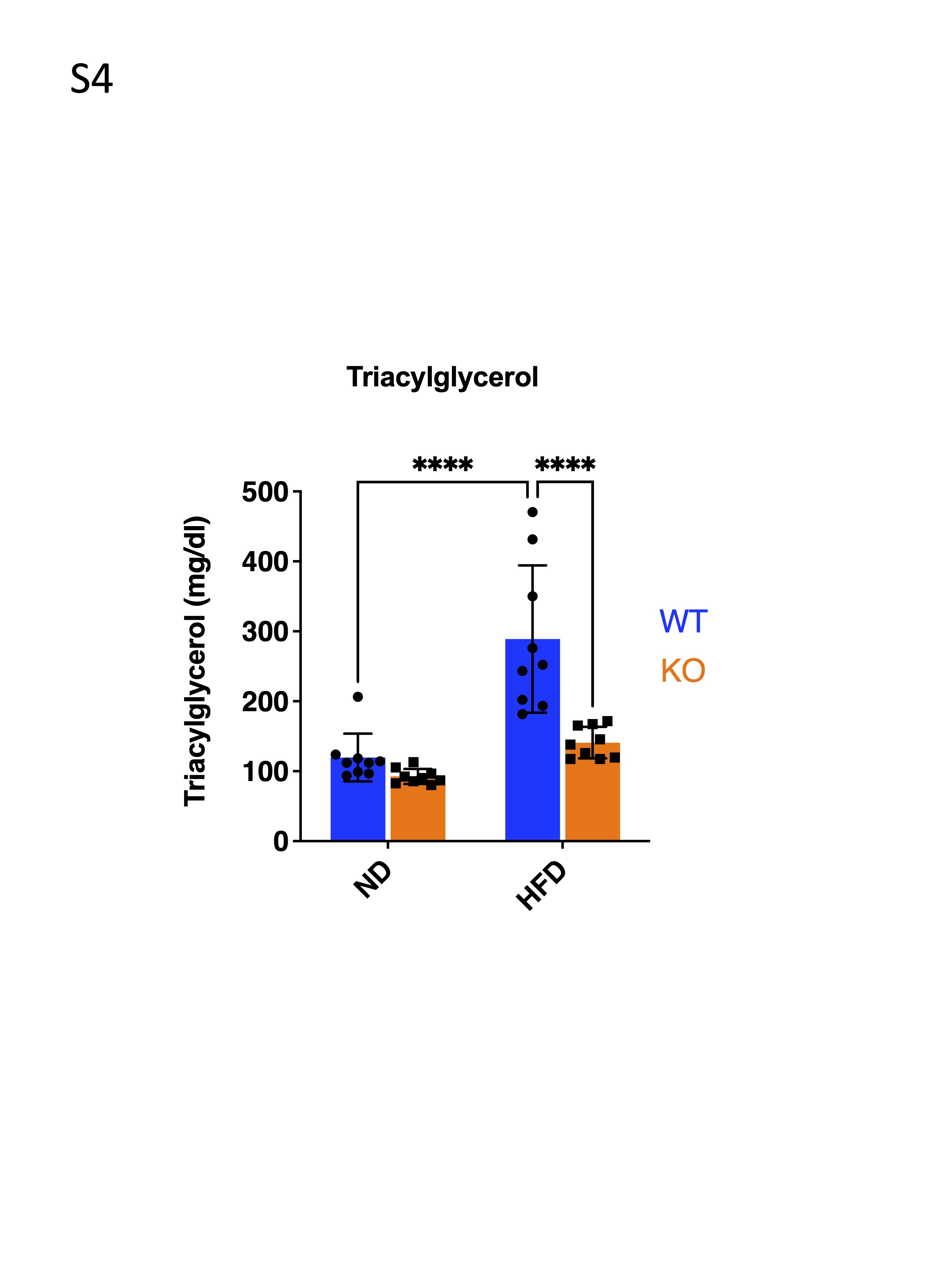


S4. Measurement of serum triacylglycerol level in ND- or HFD-fed WT and Lrrk2-KO mice.

We measured triacylglycerol level in mice serum after 5 months of feeding ND or HFD using LabAssay Triglyceride kit (FUJIFILM Wako, Tokyo, Japan), as followed manufacturer's instructions. Data are presented as means ± SEM (n = 9). The data were analyzed by two-way ANOVA combined with Tukey’s post hoc test. ****p<0.0001 (WT vs KO).


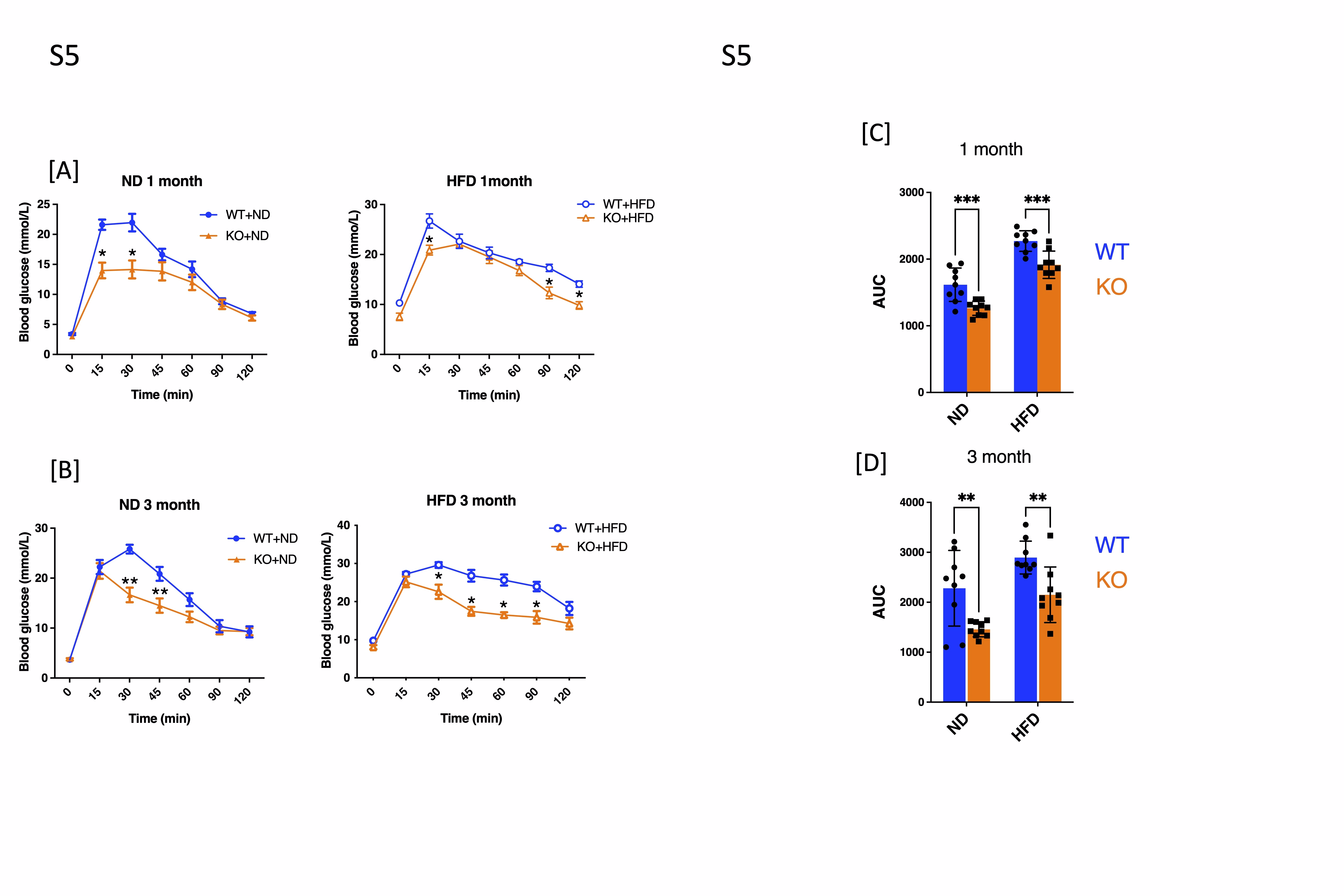


S5. Comparison of blood glucose changes in OGTT of ND- or HFD-fed WT and Lrrk2-KO mice.

Five-week old WT and Lrrk2-KO mice were reared on ND or HFD for 20 weeks. OGTT was performed at 1 and 3 months from the start of feeding each diet. Blood glucose variation curve of ND group (left panel) and HFD group (right panel) at 1 (A) and 3 (B) months are shown with wild type animals in blue and Lrrk2-knockout in red. Area under the curve (AUC) of OGTT at 1 (C) and 3 (D) months were indicated. Data are presented as means ± SEM (n = 9 animals per group). Data of blood glucose variation curve were analyzed by two-way ANOVA combined with Sidak post hoc test. *p<0.05, **p<0.01, ***p<0.001 (WT vs KO).


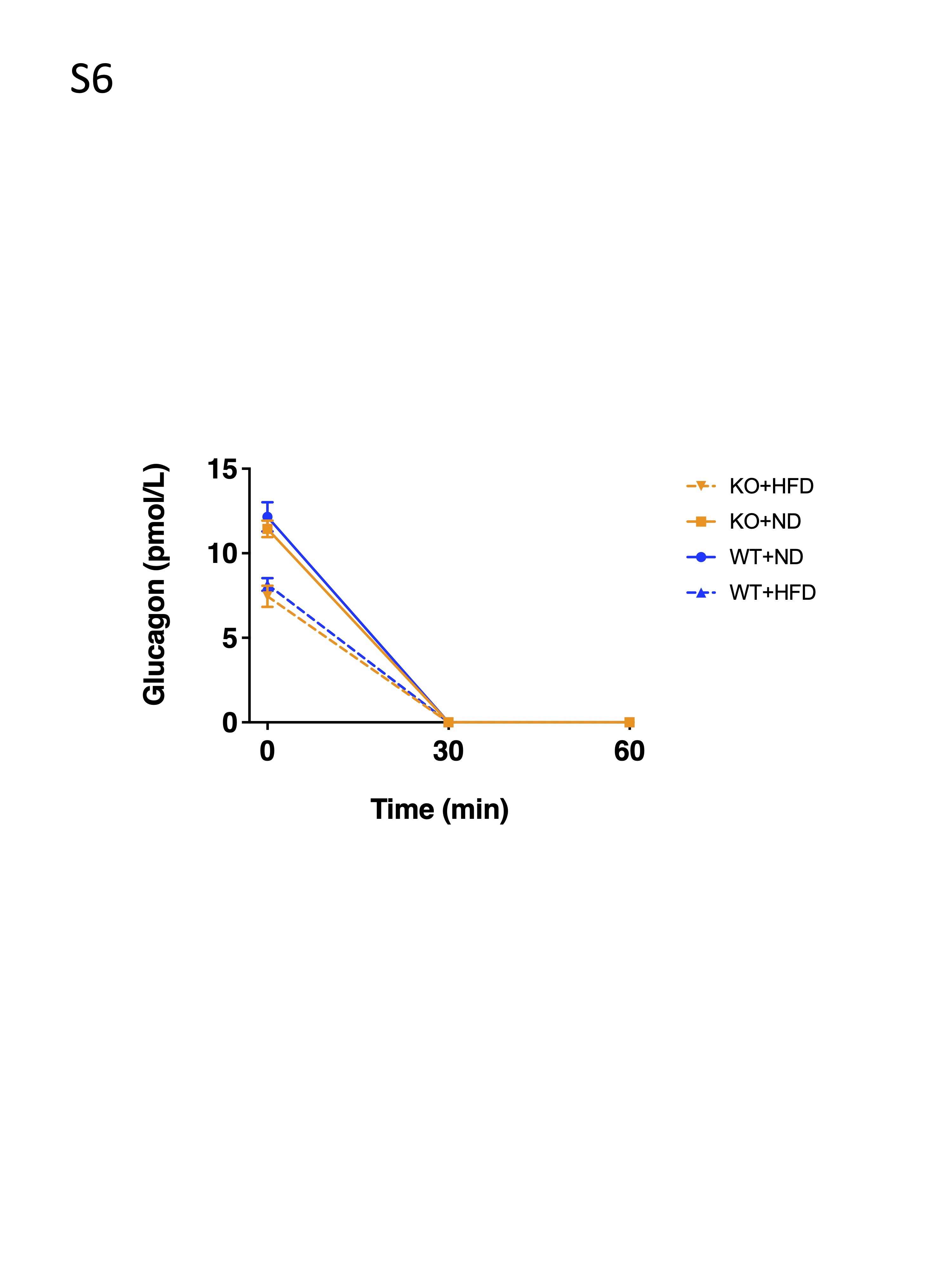


S6. Comparison of serum glucagon levels of ND- or HFD-fed WT and Lrrk2-KO mice.

Time course of serum glucagon levels in glucose injection at 5 months of ND-fed WT, Lrrk2-KO mice and HFD-fed WT, KO mice were measured by ELISA. Data are presented as means ± SEM (n = 9 animals per group). The data were analyzed by two-way ANOVA combined with Tukey’s post hoc test.


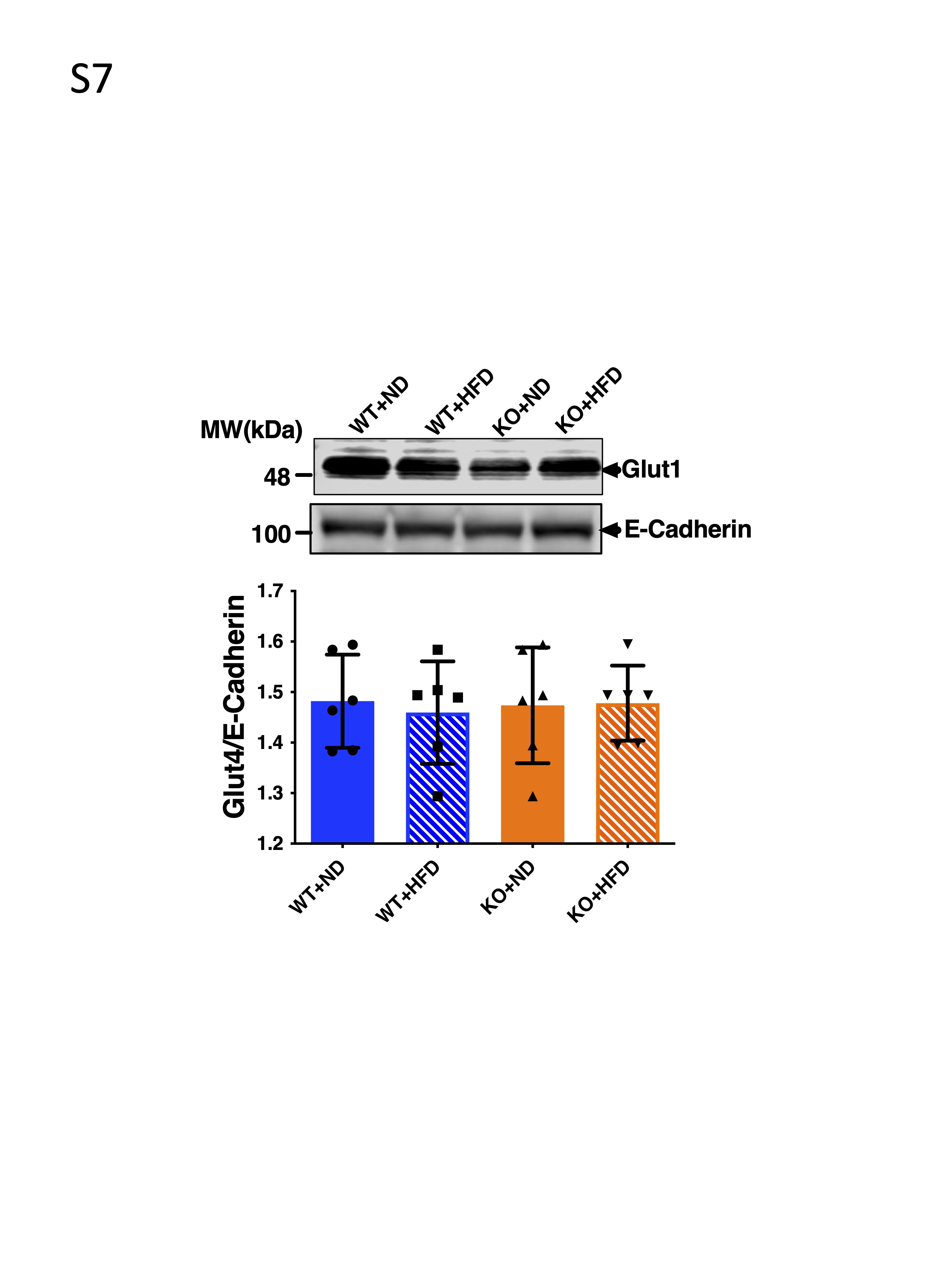


S7. GLUT1 contents in PM fraction prepared from adipose tissue of ND- or HFD-fed WT and Lrrk2-KO mice.

GLUT1 contents in PM fraction was determined by western blotting with an anti-GLUT1 antibody. The intensity of Glut4 bands was normalized by E-cadherin. Data are presented as means ± S.D. (n = 3). The data were analyzed by one-way ANOVA combined with Tukey’s post hoc test.


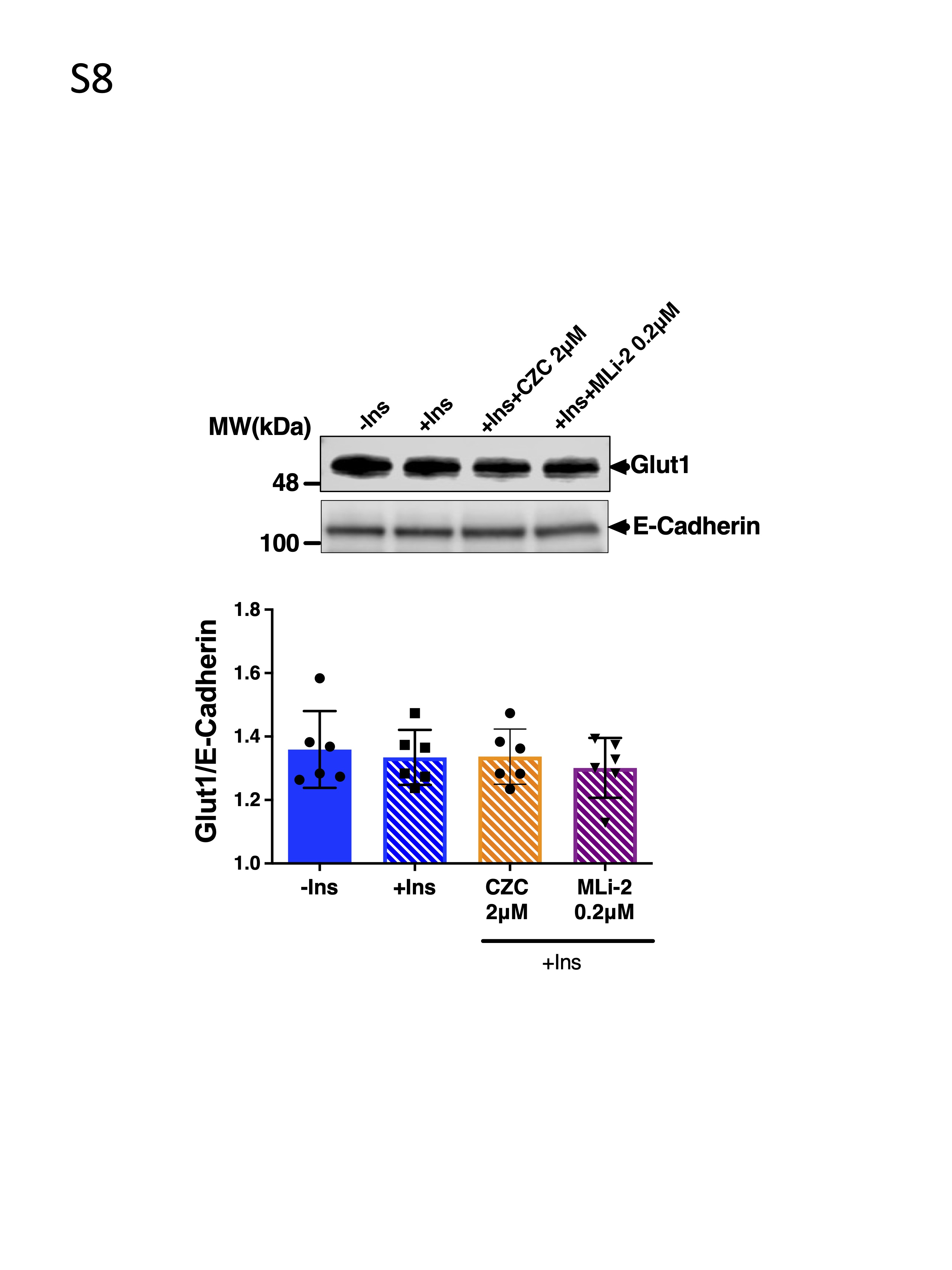


S8. GLUT1 contents in PM fraction prepared from adipocyte.

Differentiated 3T3-L1 adipocytes were serum starved and were then treated with or without CZC25146 (2 µM) and MLi-2 (0.2 µM). After that, the cells were stimulated with insulin (100 nM) for 30 min. Quantification of Glut1 in the plasma membrane (PM) fraction obtained from differentiated 3T3-L1 adipocyte after stimulation by 100nM insulin with or without LRRK2 inhibitors by western blotting with an anti-GLUT1 antibody. The intensity of Glut11 bands was normalized by E-cadherin. Data are presented as the mean±S.D. (n=6). The data were analyzed by one-way ANOVA combined with Tukey’s post hoc test.


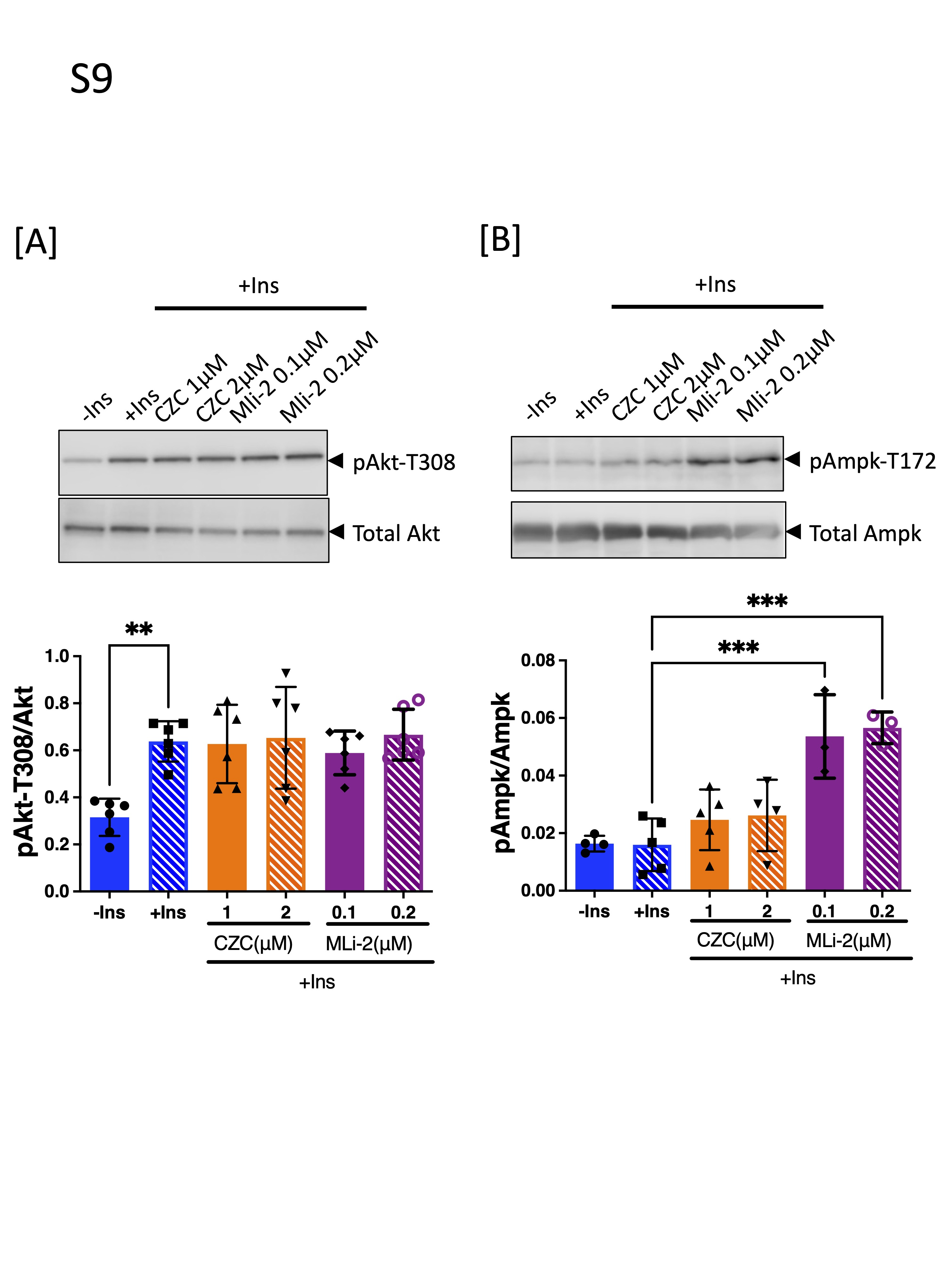


S9. Effect of LRRK2 kinase inhibitor on the phosphorylation of Akt and Ampk in adipocyte.

3T3-L1 cells treated with or without CZC25146 (1 and 2 µM) and MLi-2 (0.1 and 0.2 µM) after serum starved and then stimulated with insulin for 30min. The cells were harvested and analyzed by western blotting using antibodies against phosphorylated or total protein such as [A] phospho-Akt (Thr308) and [B] phospho-Ampk (Thr172) The expression level of phosphorylated protein was normalized against the total expression level of the target protein. Data are presented as means ± S.D. (n = 6). The data were analyzed by one-way ANOVA combined with Tukey’s post hoc test. **p<0.01, ***p<0.001.


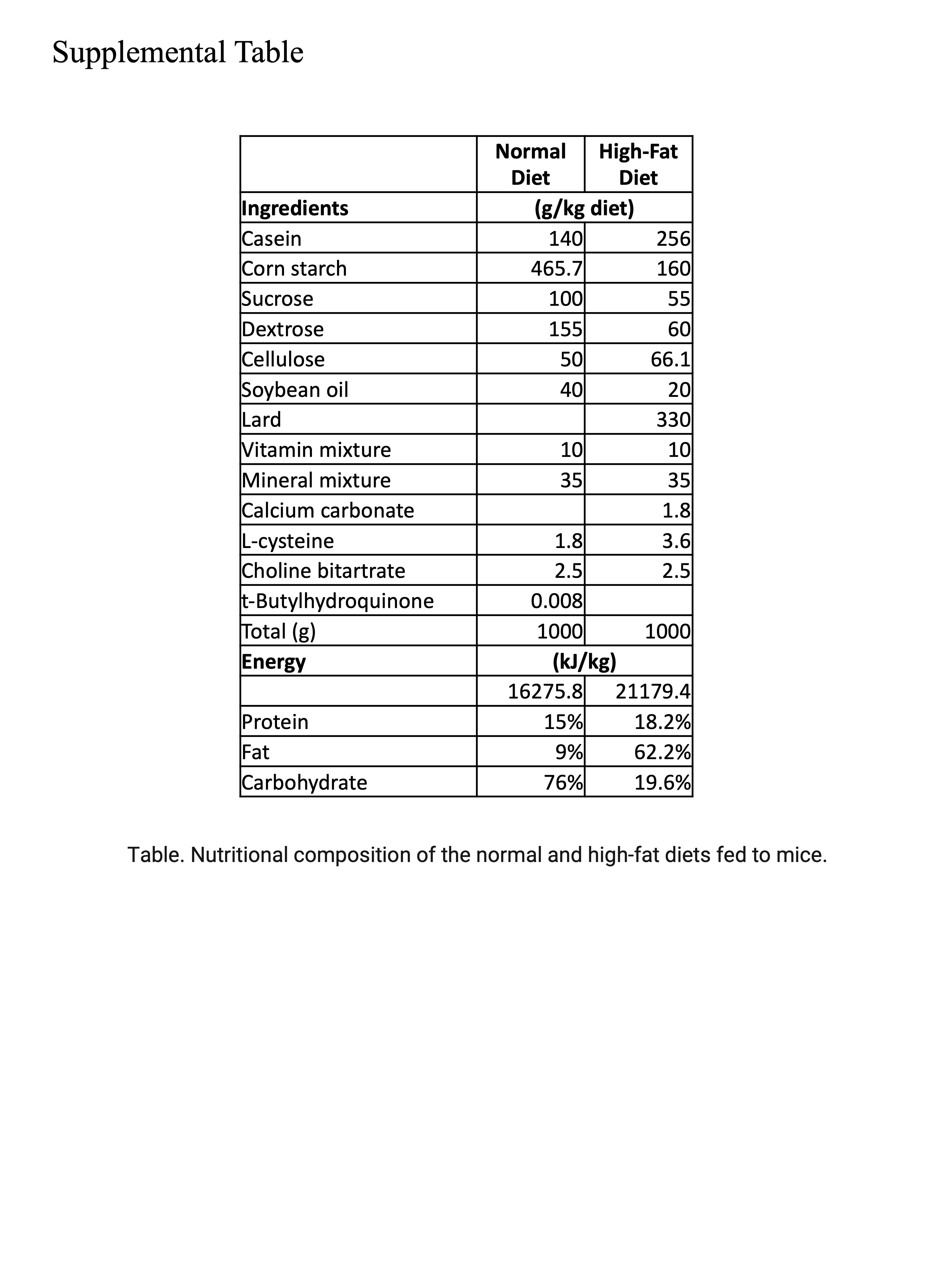


Table S1. Nutritional composition of the normal and high-fat diets fed to mice.
